# Antibiotic use in oyster hatcheries promotes rapid spread of a highly transferable and modular resistance plasmid in *Vibrio*

**DOI:** 10.1093/ismejo/wraf163

**Published:** 2025-08-13

**Authors:** Julia Mougin, Yannick Labreuche, Viviane Boulo, David Goudenège, Jamal Saad, Gaelle Courtay, Jacqueline Le Grand, Oriane Chevalier, Juliette Pouzadoux, Caroline Montagnani, Marie-Agnès Travers, Bruno Petton, Delphine Destoumieux-Garzón

**Affiliations:** IHPE, Université de Montpellier, CNRS, IFREMER, Université de Perpignan Via Domitia, place E. Bataillon, 34090 Montpellier, France; IHPE, Université de Montpellier, CNRS, IFREMER, Université de Perpignan Via Domitia, place E. Bataillon, 34090 Montpellier, France; IHPE, Université de Montpellier, CNRS, IFREMER, Université de Perpignan Via Domitia, place E. Bataillon, 34090 Montpellier, France; IFREMER, IRSI, SeBiMER Service de Bioinformatique de l’Ifremer, ZI de la Pointe du Diable, 29280 Plouzané, France; IHPE, Université de Montpellier, CNRS, IFREMER, Université de Perpignan Via Domitia, place E. Bataillon, 34090 Montpellier, France; IHPE, Université de Montpellier, CNRS, IFREMER, Université de Perpignan Via Domitia, place E. Bataillon, 34090 Montpellier, France; IFREMER, Unité écoPHYsiologie et Traits d'histoire de vie des orgaNismES marinS (PHYTNESS), ZI de la Pointe du Diable, 29280 Plouzané, France; Laboratoire des sciences de l'environnement marin (LEMAR), Université de Bretagne Occidentale, CNRS, Institut de recherche pour le développement (IRD), Ifremer, Technopôle Brest-Iroise, Rue Dumont D'urville, 29280 Plouzané, France; IHPE, Université de Montpellier, CNRS, IFREMER, Université de Perpignan Via Domitia, place E. Bataillon, 34090 Montpellier, France; IHPE, Université de Montpellier, CNRS, IFREMER, Université de Perpignan Via Domitia, place E. Bataillon, 34090 Montpellier, France; IHPE, Université de Montpellier, CNRS, IFREMER, Université de Perpignan Via Domitia, place E. Bataillon, 34090 Montpellier, France; IHPE, Université de Montpellier, CNRS, IFREMER, Université de Perpignan Via Domitia, place E. Bataillon, 34090 Montpellier, France; IFREMER, Unité écoPHYsiologie et Traits d'histoire de vie des orgaNismES marinS (PHYTNESS), ZI de la Pointe du Diable, 29280 Plouzané, France; Laboratoire des sciences de l'environnement marin (LEMAR), Université de Bretagne Occidentale, CNRS, Institut de recherche pour le développement (IRD), Ifremer, Technopôle Brest-Iroise, Rue Dumont D'urville, 29280 Plouzané, France; IHPE, Université de Montpellier, CNRS, IFREMER, Université de Perpignan Via Domitia, place E. Bataillon, 34090 Montpellier, France

**Keywords:** antibiotic resistance, *Vibrio* spp, oyster, hatchery, pAQU plasmid, conjugative transfer, AMR, MDR, shellfish farming

## Abstract

Plasmids play a key role in the horizontal gene transfer of antibiotic resistance genes (ARGs), particularly in aquaculture where ARG-carrying *Vibrio* bacteria are frequently detected. Given the expansion of global aquaculture and its reliance on antibiotics, we investigated how these practices influence the emergence, dynamics, and spread of ARGs, focusing on *Magallana gigas* hatcheries—the world’s most widely farmed shellfish. Among the three antibiotics tested, only chloramphenicol (CHL) led to a pronounced selection and dissemination of CHL-resistant *Vibrio* isolates. Resistance was mediated by *catA2*, located in a highly modular, insertion sequence- and transposase-rich region of a conjugative plasmid, alongside *tet(B)*. This plasmid was closely related to emerging pAQU-type plasmids unreported in Europe. pAQU-MAN, derived from Marine ANtimicrobial resistance, is a low-copy, highly transferable plasmid that rapidly spread throughout the hatchery following CHL treatment. Though naturally found in commensal *Vibrio*, it exhibited a broad host range, transferring efficiently to both oyster- and human-pathogenic *Vibrio* strains, as well as to *Escherichia coli*, with high conjugation rates. Additionally, it remained stable in *Vibrio* hosts and was transmitted from oyster parents to progenies, even in the absence of antibiotic. It eventually disappeared from the microbial community associated to adults. Our findings highlight that antibiotic use in oyster hatcheries can select for highly modular and transferable multidrug-resistant plasmids, posing a risk of environmental dissemination, although their limited persistence in juvenile oyster reduces the likelihood of transmission to humans. We discuss the human and ecological factors driving pAQU-MAN spread and control in aquaculture settings.

## Introduction

Widespread antibiotic overuse has fueled the global rise of antimicrobial resistance (AMR), which is widely recognized as a major global threat to human, animal, plant, and ecosystem health [1]. AMR arises through genetic mutations and horizontal gene transfer (HGT) involving mobile genetic elements (MGEs) carrying antibiotic resistance genes (ARGs), such as transposons, insertion sequences (ISs), and plasmids [2]. In particular, conjugative plasmids—transferring genetic material between donor and recipient cells by physical contact—can drive the spread of antibiotic resistance across diverse environments and bacterial taxa [3]. This is exemplified by the global dissemination of plasmid-encoded *mcr-1* from livestock to humans, undermining the efficacy of colistin, a last-resort antibiotic, and heightening the risk of therapeutic failure [4, 5].

Different sectors of activity have been implicated as contributors to the global spread of antibiotic resistance, including aquaculture [6, 7]. With a rising global population and declining wild seafood stocks, aquaculture has become crucial for food security. Over the past 20 years, global aquaculture production has steadily increased, with 75% of it dominated by carps, tilapia, catfish, seaweeds, and bivalves [8]. Infectious diseases like vibriosis pose a major challenge in aquaculture, leading to heavy antibiotic use. Although some countries have reduced reliance through vaccination and improved practices, antibiotics remain essential for treatment and are often used preventively [9]. The oyster industry is of particular concern, as oysters are the most widely farmed mollusk globally and are often consumed raw, which heightens the risk of ingesting antibiotic-resistant bacteria (ARBs), including *Vibrio* species pathogenic to humans [10, 11]. This raises significant concerns because *Vibrio* bacteria are currently considered a key global and emergent public health risk [11]. Different studies have raised concerns about antibiotic-resistant *Vibrio* pathogens in oysters [12–14]. For instance, in a commercial aquaculture facility in the USA, *Crassostrea virginica* were found to harbor potential *Vibrio* species pathogenic to humans, such as *Vibrio parahaemolyticus* and *V. vulnificus*. These isolates exhibited resistance to several antibiotics, including cephalosporins and tetracyclines (TETs), which are recommended by the US Centers for Disease Control and Prevention for treating *Vibrio* infections in humans [14]. Understanding the key drivers of AMR in aquaculture is therefore crucial for developing effective mitigation strategies under the “One Health” approach, which recognizes the interconnected health of humans, animals, and the environment [15]. A critical gap in knowledge remains in understanding how different veterinary antibiotics contribute to AMR in *Vibrio* bacteria—a challenge further complicated by significant variations in antibiotic regulations across countries.

*Vibrio* bacteria are known as reservoirs of plasmid-borne resistance [16]. Thus, *V. splendidus* was implicated in the transfer of quinolone resistance in marine environments [17]. Similarly, *V. parahaemolyticus* carrying NDM-1 carbapenemase resistance, capable of transfer to *Escherichia coli*, has been detected in imported shrimp from Vietnam to France, underscoring a potential global pathway for resistance dissemination [18]. Alarmingly, plasmid-borne resistance can propagate in shellfish hatcheries, as demonstrated in research facilities in Spain after the use of sublethal antibiotic concentrations, although the specific plasmid involved remains uncharacterized [19]. However, the human and environmental cues governing the spread and persistence of plasmid-associated AMR in *Vibrio* bacteria are still poorly understood. Such fundamental questions about plasmid epidemiological dynamics have been addressed in a number of non-*Vibrio* human pathogens [20], mostly in laboratory conditions. This has prompted recent reviews to call for studies on plasmid dynamics that better reflect real-world situations, a knowledge gap particularly critical in aquaculture, where the emergence of AMR in *Vibrio* bacteria not only undermines the treatment of vibriosis but also poses a serious public health risk [3, 21].

The objective of this study was to assess the impact of antibiotics on the development, dynamics, and spread of ARGs in *Vibrio* populations circulating in hatcheries of *Magallana gigas* oysters—the main mollusk species cultured worldwide—with a particular interest in plasmid-mediated AMR. This controlled but ecologically representative aquaculture system provided a relevant setting to investigate how antibiotic use influences microbial ecology, with direct implications for both oyster health and potential human exposure. In oyster hatcheries, bacterial diseases often cause mortalities during two critical phases: broodstock gametogenesis—when rising temperatures and increased feeding boost bacterial loads—and early larval development, when oysters are highly vulnerable to infection. To manage these mortalities, antibiotic use can be implemented to varying degrees depending on national regulations and the specific objectives of the hatchery, whether for commercial production or research. In this study, we tested three veterinary antibiotics classified by the World Health Organization as highly or critically important for human medicine [22], applying them under a worst-case prophylactic scenario to assess their potential impact on antimicrobial resistance. (i) chloramphenicol (CHL): although efforts have been made to ban this antibiotic for food production in many countries worldwide, it still seems to surface in some continents [6, 9, 23, 24]. CHL-resistant *Vibrio* pathogens contributed to larval culture failures of clam species in an experimental shellfish hatchery in Spain; resistance was plasmid-encoded [19]. (ii) Florfenicol (FLO): a monofluorinated derivative of CHL authorized for use in food-producing animals in most countries worldwide [9, 25]. FLO was shown to favor the emergence of FLO-resistant genes in channel catfish tanks, though its impact on *Vibrio* bacteria remains unexplored [26]. (iii) Flumequine (FLU): a fluoroquinolone widely used in aquaculture, which is mainly employed against Gram-negative bacteria [27]. It has been associated with the development of resistance in *Aeromonas* pathogens affecting rainbow trout in France [28].

Here, oysters with six distinct antibiotic treatment histories were produced in the experimental hatchery. The development of AMR in *Vibrio* isolates was monitored in seawater and oyster tissues under different antibiotic conditions. The oyster resistome and mobilome profiles were analyzed through pool sequencing of bacterial genomes. The dynamics of ARGs at different times of the hatchery process were assessed by quantifying their abundance in oyster tissues. Finally, to explore the spread of resistance genes and plasmids across bacterial species, associated ARGs were genetically characterized using whole genome sequencing of bacterial isolates. Our study shows that CHL usage has long-lasting transgenerational effects on the resistome of oysters. It induces the rapid spread of CHL resistance through the propagation of a highly transferable, broad-host-range conjugative plasmid, hosting an IS-rich platform for ARG exchange. This newly discovered plasmid belongs to the pAQU family repeatedly isolated in *Vibrionaceae*.

## Materials and methods

### Antibiotic treatment and sample collections

**Figure 1:**
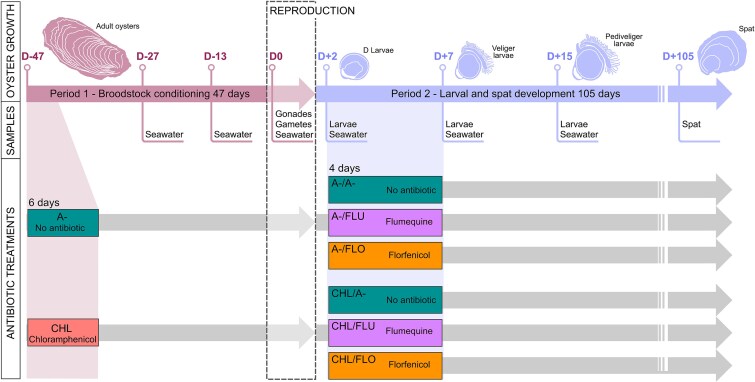
Experimental design. The rearing of *Magallana gigas* consisted of two main periods: Period 1 focused on broodstock conditioning, essential for gametogenesis and culminating in reproduction, while Period 2 involved larval growth until the spat stage. At the start of Period 1, the broodstock was divided into two groups based on treatment conditions: one group received chloramphenicol (CHL), whereas the other was untreated (A−), with two tanks allocated for each condition. After reproduction, the larvae from both the CHL-treated and A-untreated broodstock were further divided into three groups: one treated with flumequine (FLU), one treated with florfenicol (FLO), and one left untreated (A−). This resulted in oysters with six distinct antibiotic histories: A−, CHL, A−/A−, A−/FLU, A−/FLO, CHL/A−, CHL/FLU, and CHL/FLO. Similarly, two replicate tanks were allocated for each condition. During Period 1, various samples, including seawater, gonads, and gametes, were collected on Day −27 (D-27), Day −13 (D-13), and the day of reproduction (D0). In Period 2, samples such as seawater, larvae, and spat were collected on Day 2 (D2) before antibiotic treatment, and on Day 7 (D + 7), Day 15 (D + 15), and Day 105 (D + 105) after antibiotic treatment.

The juvenile production of *Magallana gigas* was conducted in a fully biosecure laboratory using ultraviolet (UV)-irradiated and filtered seawater, with treated effluents to ensure antibiotic removal. It was performed in two distinct phases, each involving specific antibiotic treatments: CHL was applied during gametogenesis, while FLU and FLO were administered post-reproduction (Fig. 1). During Phase 1 (47 days), adult oysters (collected from the natural environment, Fouras, France) underwent broodstock conditioning required for gametogenesis, culminating in reproduction. Oysters were allocated into four 400 l tanks. Two tanks received chloramphenicol (CHL; 8 mg·l^−1^) administered in three 48-h baths over 6 days—conditions known to effectively control bacteria in oyster tissues [29–31]. Two tanks served as controls (A−). At Day 0, gametes were separately harvested from each treatment and pooled for fertilization. During Phase 2 (105 days), embryos were reared to the spat stage. On Day 2 of larval development, progenies resulting from CHL-treated and control broodstock were divided into three groups: one received no antibiotics, another was treated with flumequine (FLU, 9.4 mg·l^−1^) administered in two repeated baths of 48 h for 4 days, and the third received florfenicol (FLO, 6.6 mg·l^−1^) under similar conditions. These antibiotic concentrations are those recommended in veterinary prescriptions for this project. Water samples (100 μl) were collected at critical time points during both phases and plated on thiosulfate–citrate–bile salts–sucrose (TCBS) agar, with or without antibiotics (CHL 10 mg.l^−1^, FLU 9.4 mg.l^−1^, or FLO 6.6 mg.l^−1^). Plates were then incubated at 20°C during 24 or 48 h, until *Vibrio* colonies appeared. Concurrently, samples from gametes, gonads, larvae (5000 individuals per sample), and spat (triplicate individuals) were homogenized and processed for both culture-based and molecular analyses. Oyster tissues for molecular analyses were stored at −80°C. At the end of each antibiotic treatment, to ensure antibiotic removal at the water outlet, filtration through an activated carbon filter was performed. Comprehensive details on antibiotic usage are provided in Supplementary Table S1; tank configuration, environmental conditions, and sample collection procedures are provided in Supplementary Material and Methods 1.1 and 1.2.

### Detection of ARGs by bacterial pool sequencing

For each condition, up to 24 colonies, grown on TCBS supplemented with CHL, were randomly selected and subcultured on Zobell agar to ensure purity. A total of 175 *Vibrio* isolates, from seawater and oyster tissue, were grouped into 12 pools according to sample types, antibiotic history, and oyster developmental stage. For pooling, individual colonies were grown overnight in liquid Zobell medium, then combined in equal volumes to create a representative pooled culture for each sample and condition. After centrifugation for 10 min at 5000 × g, genomic DNA was extracted from the pellet using the NucleoSpin Tissue kit (Macherey-Nagel), with DNA quality and concentration verified via NanoDrop and Qubit assays. Sequencing libraries were constructed using the Nextera XT DNA Library Prep Kit (Illumina) after adjusting the DNA concentration to 1 ng.μl^−1^. Equimolar libraries were sequenced on a NextSeq 550 (Illumina, paired-end, 2 × 150 bp) at the BioEnvironment platform of the University of Perpignan Via Domitia (UPVD, https://bio-environnement.univ-perp.fr/). Sequences assemblies and quality check was performed on a home-based cluster DATARMOR, the Ifremer’s supercomputer. Sequence reads were quality-checked using FastQC (v0.11.9) and trimmed with Trim Galore (v0.6.7) [32]. *De novo* assembly was performed with metaSPAdes (v3.15.4) [33] and assessed with QUAST (v5.0.2) [34]. Contigs were classified using PlasFlow (v1.1.0) into plasmid-like, chromosome-like, or unidentified sequences [35]. ARGs were identified with ABRicate (v1.0.1) against the ResFinder [36] and Comprehensive Antibiotic Resistance Database (CARD) [37] databases (minimum 65% coverage and identity) [38]. Metagenomes were annotated with Prokka (v1.14.6) [39], and ARG sequences were verified via Basic Local Alignment Search Tool (BLAST) against GenBank. Incomplete ARG sequences were discarded, and the remaining sequences were aligned using MEGA11 (ClustalW) [40]. The phylogenetic tree was constructed using the Neighbor-Joining method with 1000 bootstrap replicates. The resulting tree was visualized and refined using iTOL (v6) [41].

### Quantification of ARGs by quantitative polymerase chain reaction (qPCR)

DNA from adult oysters (collected on Day 0, Fig. 1) and larvae (collected on Days 2, 7, and 15, Fig. 1) was prepared as described above. Primers were designed to target the CHL resistance genes (*catA2*; see Table S2) and were used to measure gene abundance in the samples, normalized to total bacterial load by quantifying the 16S ribosomal RNA (rRNA) genes with Total_B_926F/Total_B_1062R primers. The qPCR reactions (1.5 μl total volume) included 1 μl SensiFAST SYBR No-ROX mix, 0.2 μM of each primer, and 0.5 μl template DNA (adjusted to 50 ng·μl^−1^). Thermal cycling was performed on a LightCycler 480 with an initial denaturation at 95°C for 3 min, followed by 40 cycles (10 s at 95°C, 20 s at 60°C, 25 s at 72°C) and a subsequent melting curve analysis. Pooled DNA, positive plasmidic controls (Eurofins Genomics), and no-template controls were included in every run, and qPCR efficiency was determined using an 8-point, 10-fold dilution series. Samples with 16S rRNA gene cycle threshold (CT) values >30 were excluded, and relative ARG abundance was expressed as the ratio of the log of absolute *catA2* and 16S rRNA gene quantifications, as previously described [42].

### Comparative analysis of plasmid genomes

Two *catA2*-positive isolates (Interactions Hôtes Pathogènes Environnements (IHPE) 6488 collected from sperm on Day 0 and IHPE 6718 collected from larvae on Day 7), differing in saccharose fermentation on TCBS, were randomly selected for Nanopore sequencing to confirm *catA2* localization on a plasmid. DNA from an overnight pure bacterial culture in Zobell medium supplemented with CHL at 20°C was extracted using the Wizard Genomic DNA Purification Kit (Promega). MinION Nanopore sequencing and assembly were performed by Eurofins Genomics (Germany). Isolate identification was based on Average Nucleotide Identity (ANI, FastANI v1.3) and core-genome analysis. Core-genome alignment (474 concatenated genes, ≥90% BLAST identity, present in >99% of genomes) was generated using Roary (v3.13.0), and a maximum-likelihood tree was constructed with IQ-TREE (v2.3.3) using 1000 bootstraps, then visualized with iTOL (v6).

For plasmid prevalence assessment, 347 isolates recovered on TCBS (without antibiotics) were screened by PCR and confirmed by sequencing. Briefly, DNA from strains positive for *catA2* and *tr1*, with 16S rRNA genes used as a quality control (see Table S2 for primer details), was extracted from 1 ml of an overnight culture in Luria Broth (LB) + 0.5 M NaCl at 20°C, using the Wizard Genomic DNA Purification Kit (Promega) and sequenced on the NextSeq System. Genome assemblies were generated with SPAdes (v4.1.0) and assessed using QUAST (v5.0.2). Plasmid scaffolding was then manually done by aligning reads against previously obtained Nanopore assemblies.

For plasmid comparison, the 27 most similar plasmids from the National Center for Biotechnology Information (NCBI) database were analyzed to assess synteny and phylogenetic relationships (see Table S3 for plasmid metadata). All plasmids were reoriented to begin at the *repA* helicase gene, in accordance with the reference plasmid pAQU1 from *Photobacterium damselae* subsp. *damselae* 04Ya311 (NC_016983.1). Gene prediction and functional annotation were performed using Bakta (v1.10.1) [43]. The ARG identification was performed using the Abricate tool with the ResFinder database, applying a threshold of 70% coverage and 80% identity, which ensures accurate identification according to established criteria [44]. A phylogenetic tree was generated with PanACoTA (v1.4.1) [45], using a 30% identity threshold for gene clustering and a 90% presence cutoff to define the persistent genome. Phylogenetic inference was then performed with IQ-TREE2 (v2.0.3) [46] under the General Time Reversible (GTR) model with 1000 bootstrap replicates. Plasmid synteny was assessed using the MMseqs2 cluster module (v15.6f452) [47], followed by ordering based on Mash distances (v2.3) [48]. Visualization was achieved with pyGenomeViz (v1.4.1) [49] and final figures were refined using Inkscape.

### Plasmid persistence assays

A 10-day plasmid persistence assay was conducted to assess the stability of the resistance plasmid in IHPE 6488 and IHPE 6718 strains. Single colonies were cultured overnight in LB + 0.5 M NaCl with CHL to ensure full plasmid retention (T0), then subcultured daily (1:100) for 10 days in media with or without CHL at 5 mg.l^−1^ (*n* = 4 for each condition). Cultures were incubated at 20°C with shaking. On Days 1, 2, 3, 5, 7, and 10, samples were collected for DNA extraction by boiling at 99°C for 10 min and then diluted 1:100 in DNase-free water and stored at −20°C. Plasmid persistence was measured by qPCR, based on the ratio of the plasmid-encoded *tr2* gene to the single-copy chromosomal *recA* gene [50]. Standard curves were generated using known quantities (∼10^6^–10^2^ molecules per reaction) of plasmids pCRTM2.1-Tr (4062 bp) and pCRTM2.1-RecA (4067 bp), constructed by cloning the *tr2* and *recA* genes into pCRTM2.1 using a TA Cloning kit. In these assays, 4 μl of diluted lysate was added to a 25 μl reaction containing LightCycler 480 SYBR Green I Master and 0.2 μM of each primer. Thermal cycling conditions were: 95°C for 10 min, 40 cycles of 95°C for 10 s, 60°C for 20 s, and 72°C for 25 s, followed by a melting curve analysis.

### Plasmid conjugation assays

Filter mating assays were conducted to assess plasmid transfer between *Vibrio* strains. Donor and recipient strains were distinguished by colony color on TCBS agar. IHPE 6488 (green colonies) was mated with *V. crassostreae* J2-9 and *V. alginolyticus* OE12 (yellow colonies), whereas IHPE 6718 (yellow colonies) was paired with *V. atlanticus* LGP32 and *V. parahaemolyticus* IFVp22 (green colonies) (Table S4). Donor and recipient overnight cultures in LB + 0.5 M NaCl were mixed 1:1, centrifuged for 5 min at 2000 × g, resuspended in 50 μl fresh medium, and applied to 0.45 μm filters on Tryptic Soy Agar (TSA) plates (with 15 g·L^−1^ NaCl). After overnight incubation at 25°C, cells were resuspended in 2 ml LB + 0.5 M NaCl and plated on TCBS agar with and without CHL at 5 mg.l^−1^, to count recipients and transconjugants. Transfer efficiency was calculated as the transconjugant-to-recipient ratio.

To confirm self-transferability, a second mating was performed using plasmid-bearing *V. atlanticus* LGP32^*^ as the donor and *V. alginolyticus* OE12 as the recipient. IHPE 6718 was also mated with kanamycin-resistant *E. coli* π3813 on TSA supplemented with thymidine (0.3 mM) to compensate recipient auxotrophy; in this case, selective plating was done on LB with thymidine and kanamycin, with or without CHL. Plasmid transfer was confirmed by colony-PCR targeting species markers and the *tr1* gene (primers in Table S2; PCR conditions in Supplementary Methods 1.3).

### Minimum inhibitory concentration assays

Minimum inhibitory concentration (MIC) assays were performed to assess the susceptibility of plasmid donor strains (IHPE 6488 and IHPE 6718) and their derived transconjugants to CHL and TET. Following CLSI VET03 and VET04 guidelines, 2-fold serial dilutions of CHL and TET (128–0.25 mg.l^−1^) were prepared from 10 g.l^−1^ ethanol stock solutions in sterile Milli-Q water [51, 52]. Ten microliters of each dilution were added to 96-well plates. Bacterial suspensions originated from an overnight culture in appropriate media of a single colony, were adjusted to a 0.5 McFarland standard in Cation-Adjusted Mueller Hinton Broth (CAMHB) (with 1% NaCl added for *V. atlanticus* LGP32). A volume of 90 μl of each suspension was inoculated per well, followed by incubation at 25°C or 35°C for 18 h with shaking at 150 rpm to ensure optimal growth for *Vibrio* bacteria. MIC values were determined visually as the lowest concentration showing no visible bacterial growth. Optical density at 600 nm was also measured using a TECAN Infinite M200 plate reader, to confirm the visual assessment and ensure reproducibility between replicates. *E. coli* CIP 76.24 (ATCC 25922) served as a control, yielding MICs within the expected CLSI ranges: 2–8 mg.l^−1^ for CHL and 0.5–2 mg.l^−1^ for TET. Sterile controls with media and antibiotics were included in each assay.

### Statistical analysis

All statistical analyses were conducted using R Studio (ver. 4.3.2). To compare the proportion of CHL-resistant *Vibrio* isolates between A− and CHL treatments, an analysis of variance (ANOVA, *F*-test) was performed, using the function AovSum. To compare the relative abundance of ARGs in larvae and gonads between A− and CHL-treated broodstock groups obtained from qPCR analysis, the Mann–Whitney test was employed due to the non-normal distribution of the data.

### Data availability

Whole-genome sequencing and pool-sequencing data of antibiotic-resistant bacteria collected at the hatchery, as part of the Spare-Sea project, are available in the European Nucleotide Archive under accession numbers PRJEB89088 and PRJEB89075, respectively.

## Results

### Use of CHL increases CHL-resistance rates in *Vibrio* bacteria isolated from the hatchery

**Figure 2:**
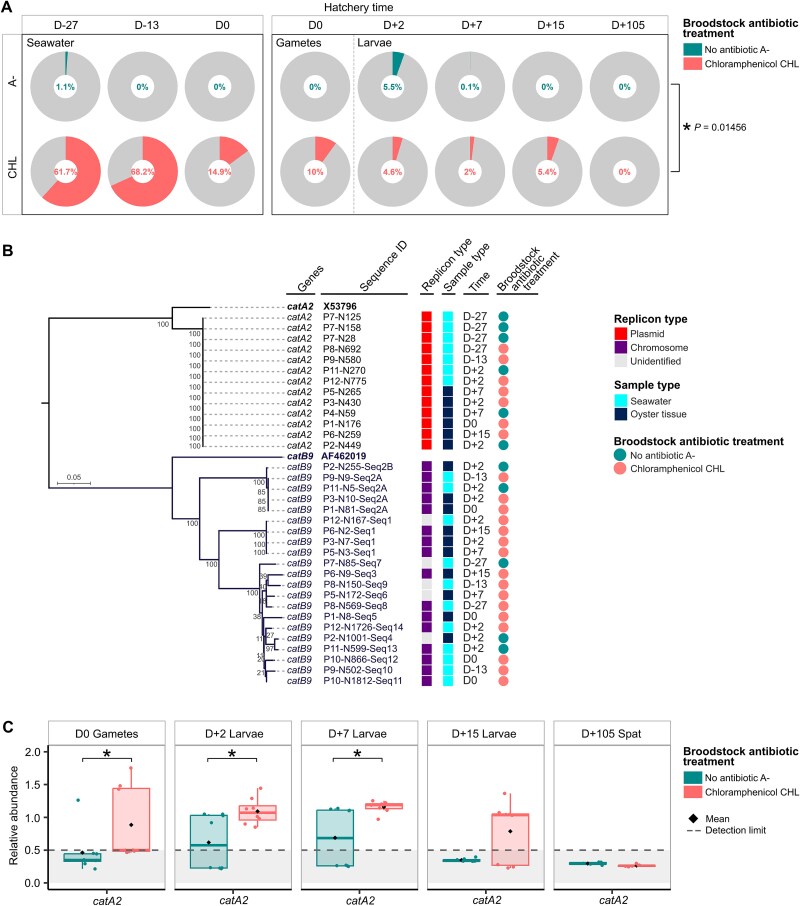
Increased prevalence of CHL-resistant *determinants* in seawater and oyster tissues following CHL treatment. (A) The percentage shown corresponds to the proportion of CHL-resistant *Vibrio* isolates—i.e., those collected on TCBS agar supplemented with CHL—relative to the total number of *Vibrio* isolates collected on TCBS agar without CHL Proportions are shown based on the treatment history of the broodstock: no antibiotic treatment (A−) and CHL treatment (CHL). Seawater samples were analyzed at Days −27 (D-27), −13 (D-13), and 0 (D0), while oyster tissues, including gametes, were analyzed on Day 0 (D0), larvae on Days 2 (D+2), 7 (D+7), and 15 (D+15) and spat on Day 105 (D + 105). Significant difference in relative abundance of CHL-resistant bacteria between A− and CHL treatment is denoted by an asterisk (^*^), as determined by the analysis of variance (ANOVA, F-test) (*P* < .05). Details of bacterial counting are provided in supplementary material Table S5. (B) *catA2* and *catB9* are widespread among putative culturable *Vibrio* strains in the hatchery, with *catA2* predicted to be plasmid-born. Midpoint-rooted phylogenetic tree of nucleotide sequences of chloramphenicol resistance genes is shown, with predictions of sequence origin: plasmid or chromosome. ClustalW and Neighbor-Joining algorithms were used, with bootstrap values calculated from 1000 replications. Two genes were identified, *catA2* and *catB9*; accession numbers of their reference sequences are depicted in bold. Sample type (seawater or oyster tissue), date of collection (from Period 1 to 2), and antibiotic treatment history are indicated on the right. Details of pool sequencing analysis and ARGs are provided in Tables S6 and S7. C. *catA2* gene persists longer in progenies from CHL-treated oysters. Relative abundance of *catA2* in both gonads, larvae, and spat samples over time were determined by qPCR. Relative abundance between samples from broodstock treated without antibiotics (A−) is depicted in green, whereas those treated with CHL are depicted in red. At Day 0 (D0) during reproduction, gonads from the broodstock were analyzed, while larvae were analyzed at Days 2 (D+2), 7 (D+7), and 15 (D+15) and spat at Day 105 (D+105). Relative abundance is expressed as the ratio of the log of absolute *catA2* and 16S rRNA gene quantifications, as previsouly described [53]. Significant differences in relative abundance of each gene between treatments A− and CHL are denoted by asterisks (^*^), as determined by the Mann–Whitney test (*P* < .05, *n* ≥ 6). Values below the detection limit indicate samples in which the ARG was not detected, whereas 16S rRNA genes were successfully amplified. Details of collected oyster tissues and sample names are provided in Table S8.

Antibiotics were applied during broodstock conditioning at D-47 and early larval development at D+2 (Fig. 1). Whereas treatments had no significant effect on oyster survival (Fig. S1), they strongly impacted the abundance of antibiotic-resistant bacteria (ARBs) in the hatchery system (Fig. 2A). During broodstock conditioning, ARBs were monitored in seawater. CHL treatment markedly increased CHL-resistant *Vibrio* rates, measured as the proportion of colonies growing on CHL-supplemented TCBS plates: 61.7% at D-27, 68.2% at D-13, and 14.9% at D0, compared to ≤1.1% in untreated controls. These elevated resistance levels persisted until reproduction at D0, up to 42 days post-CHL treatment.

During larval development, ARBs were tracked directly in oyster tissues. CHL-resistant *Vibrio* bacteria were detected in gametes and larvae from CHL-treated broodstock: 10% at D0, 4.6% at D+2, 2.0% at D+7, and 5.4% at D+15, before disappearing by D+105. In the control group, no resistant bacteria were found in gametes, and CHL-resistant *Vibrio* bacteria dropped from 5.5% at D+2 to undetectable levels by D+15. CHL exposure thus significantly increased the proportion of CHL-resistant *Vibrio* bacteria in both water and oyster tissues (ANOVA, *F*-test, *P* = .01456). In contrast, FLO and FLU had no detectable impact under our conditions, as no resistant *Vibrio* bacteria were recovered on selective media. No CHL-resistant strains were detected in the incoming seawater, indicating that resistant *Vibrio* bacteria likely originated from treated broodstock and/or seawater within the system.

### CHL-resistance genes are widespread in culturable *Vibrio* bacteria from the hatchery

We analyzed the resistome of CHL-resistant *Vibrio* bacteria isolated throughout the hatchery cycle (Fig. 2B). Pool sequencing revealed several ARGs, including *dfrA6*, *qnrS2*, *tet(34)*, *tet(35)*, *tet(B)*, *catA2*, and *catB9*. Among CHL-resistance genes, *catA2* sequences were 100% identical to each other and showed 89.6% identity and 100% coverage to the reference sequence (X53796). In contrast, *catB9* sequences were more divergent, displaying variable similarity both to each other and to the reference (53%–59% identity, 67%–89% coverage; AF462019). Both genes were consistently detected from D-27 to D+15, across seawater and oyster samples, regardless of treatment. Long-read sequencing confirmed that *catA2* was plasmid-encoded and *catB9* chromosomal.

### Parental CHL-treatment has long-lasting effects on the progeny resistome

We then assessed *catA2* prevalence in oyster tissues during larval development using qPCR (Fig. 2C). On D0, *catA2* was significantly more abundant in gonads from CHL-treated broodstock (*P* = .0054, Mann–Whitney test). This difference persisted in larvae at D+2 (*P* = .0281) and D+7 (*P* = .0070), with *catA2* detectable only in CHL progeny by D+15. By D+105, no signal was detected. These results show that CHL exposure leads to a prolonged enrichment of plasmid-borne *catA2* in early oyster development, with a gradual clearance over time.

### *catA2* colocalizes with *tet(B)* on a conjugative pAQU-type plasmid termed pAQU-MAN

**Figure 3:**
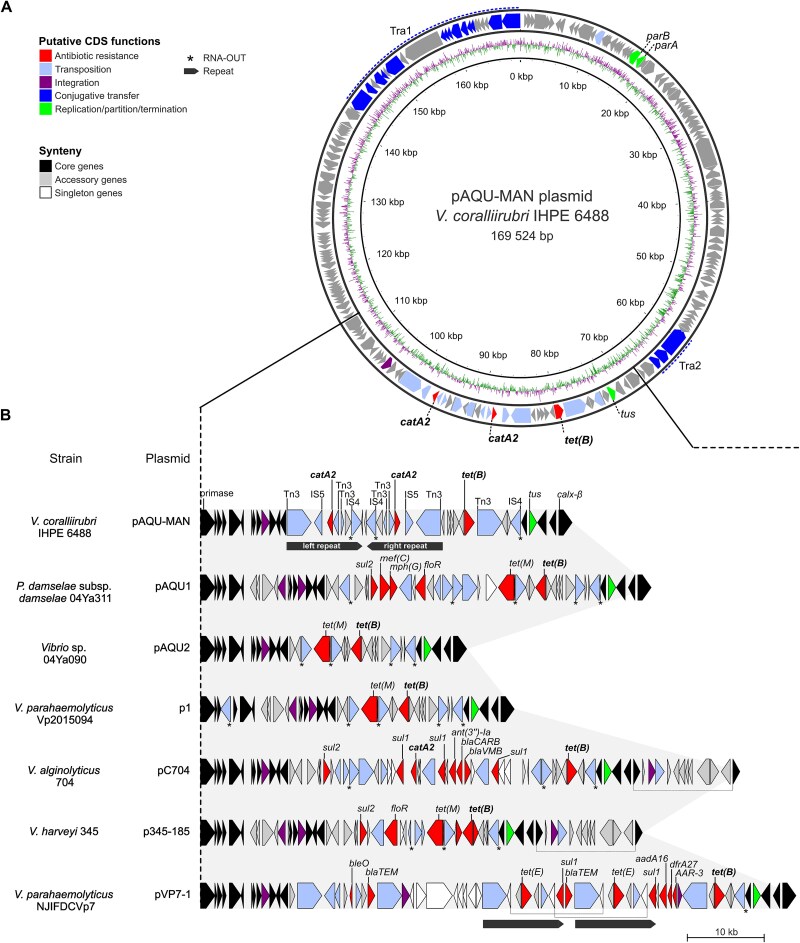
Chloramphenicol resistance genes, along with tetracycline resistance genes, are located on a pAQU-type plasmid, named pAQU-MAN (plasmid AQUatic – Marine ANtimicrobial resistance). (A) The circular map of the pAQU-MAN plasmid shows a transposase-rich region with a duplicated *catA2* in an inverted repeat, along with *tet(B)*. From the inside to the outside: (i) guanine cytosine (GC) content and (ii) putative CDS functions of the pAQU-MAN plasmid. Two regions were rich in *tra* genes, Tra1 and Tra2; *parA/parB* correspond to the partition system, and *tus* encodes for the replication terminus site-binding protein. (B) Zoomed-in view of the variable region, likely highlighting a hotspot for gene exchange. The synteny plots show structural variation among pAQU-MAN and six closely related pAQU-type plasmids. Clustering was performed using MMseqs2 with 30% identity and 50% coverage thresholds, resulting in 153 core, 77 accessory, and 115 singleton genes across the full-length plasmids. In pAQU-MAN, *catA2* is duplicated and flanked by transposase Tn3 and insertion sequences IS4 and IS5, all located within a predicted inverted repeat (IR) region. Full genome annotations and ARG details are provided in Tables S10 and S11.

We focused our efforts on the plasmid-encoded *catA2* gene because its well-documented role in CHL (and fusidic acid) resistance made it the most likely candidate underlying CHL resistance in our *Vibrio* isolates [24, 54]. In contrast, the chromosomal *catB9* gene—previously reported as unrelated to CHL resistance in *Vibrio* bacteria [55–57]—was not investigated further. The plasmid was sequenced from two hatchery isolates, *V. coralliirubri* IHPE 6488 and *V. cyclitrophicus* IHPE 6718—both members of the Splendidus clade—as determined by core genome phylogenetic analysis and ANI value (Fig. S2, Table S9). Its sequence was nearly identical (>99.99% similarity) in the two strains with a size ranging from 169 524 bp to 170 776 bp in the *V. coralliirubri* and *V. cyclitrophicus strains,* respectively (Fig. S3). The *catA2-*carrying plasmids were predicted to be conjugative, as inferred by the presence of the *tra* machinery system (Fig. 3A). Other important plasmid features included a replication/partition/termination system including *parA*/*parB*, several transposases and insertion sequences, and antibiotic resistance genes, notably two *catA2* genes and one *tet(B)* gene (Fig. 3A, Table S10).

Plasmids carrying *catA2* belonged to the pAQU-type family, showing high similarity (>85% coverage, >99.80% identity) to known pAQU plasmids such as pAQU1 (AP026782.1), pAQU2 (AB856327.1), pVP7-1 (CP150866.1), pC704 (OP958859.1), p1 (CP080480.1), and p345-185 (CP025539.1), all from *Vibrio* or *Photobacterium* spp. Core genome and synteny analysis of 27 related plasmids from the literature confirmed a highly conserved backbone among pAQU types (Fig. S4, Table S3), with pAQU-MAN showing closest similarity to pAQU1 and pAQU2 (Fig. S4). These plasmids diverged in a region enriched in ARGs, transposases, and insertion sequences (Fig. 3B), suggesting a hotspot for gene exchange. In pAQU-MAN, this region contained two identical *catA2* genes within an inverted repeat (IR) sequence. We named this plasmid pAQU-MAN, for plasmid AQUatic Marine ANtimicrobial resistance.

**Figure 4:**
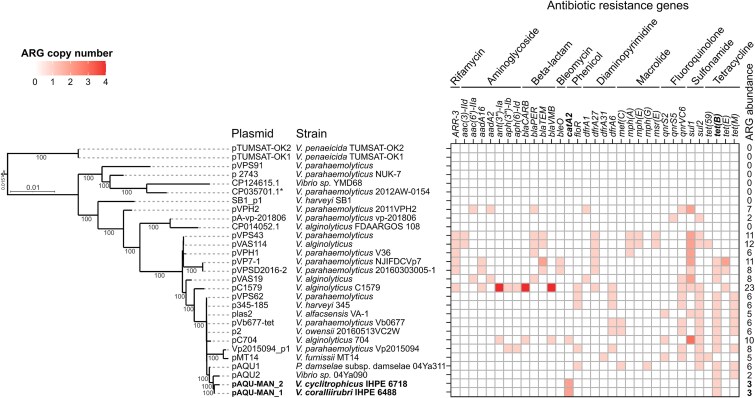
pAQU-MAN belongs to emerging multi-drug-resistant pAQU-type plasmids in the aquatic environment. Phylogenetic tree based on core genome analysis (111 gene families) from 27 closely related plasmids identified in *Vibrio* and *Photobacterium* strains. The two pAQU-MAN plasmids identified in this study are highlighted in bold. Core genome was defined using PanACoTA with a 30% identity threshold and 90% persistence, and resulted in 107 core, 314 accessory, and 278 singleton genes. The tree was constructed with IQ-TREE using the GTR model and 1000 bootstrap replicates. To the right, a heatmap illustrates both the presence/absence and the number of copies of ARGs within these plasmids, categorized by the antibiotic classes they confer resistance to. Results were obtained using the Abricate tool with the ResFinder database, applying a threshold of 70% coverage and 80% identity, which ensures accurate identification according to established criteria [44].

We then focused on this important recombination hotspot, which may drive the exchange and spread of ARGs under selective pressure. Varying numbers of ARGs, ranging from 0 and up to 23, were found on publicly available pAQU-type plasmids (Fig. 4, right panel, Table S11), indicating that this plasmid family has the potential to confer resistance to a broad spectrum of antibiotics, including rifamycin, aminoglycosides, beta-lactams, bleomycin, phenicols, diaminopyrimidines, macrolides, fluoroquinolones, sulphonamides, and tetracyclines. pAQU-MAN shared the *catA2* gene with pC704 only, whereas *tet(B*) was present in 14 out of 29 plasmids. Some closely related plasmids contained different sets of ARGs. Only the more divergent plasmids (e.g. pTUMSAT-OK1, pTUMSAT-OK2, pVPS91, p2743, SB1_p1) lacked ARGs entirely.

### pAQU-MAN spread rapidly in five *Vibrio* species within the hatchery

**Figure 5:**
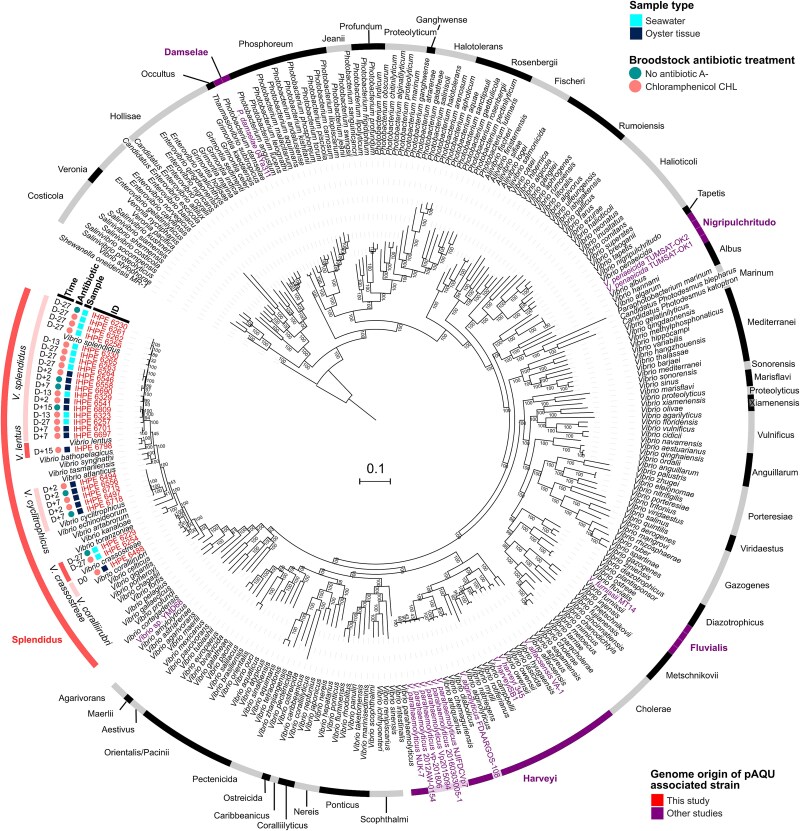
pAQU-MAN circulates between five *Vibrio* species in the hatchery. Phylogenetic distribution of pAQU-MAN-carrying isolates among 231 representative Vibrionaceae genomes, species is shown. The 27 genomes carrying pAQU-MAN, identified in this study are highlighted. They all belong to the *Vibrio* clade Splendidus, specifically to species such as *V. splendidus*, *V. lentus*, *V. cyclitrophicus*, *V. crassostreae*, and *V. coralliirubri*. Sample type (seawater or oyster tissue), collection period, and the antibiotic treatment history of the broodstock are also indicated. In addition, fifteen Vibrionaceae genomes previously identified in the literature and available in public databases are shown, all containing pAQU-like plasmids. Pangenome analysis using PanACoTA v1.4.0 (75% identity, 90% persistence) identified 491 persistent gene families. The tree was inferred with IQ-TREE2 (v2.3.6, GTR model, 1000 ultrafast bootstraps), and rooted using *Shewanella oneidensis* MR-1 (GCF_000146165.2). Details of previously published genome sequences in Table S12.

We further asked whether pAQU-MAN could have spread between *Vibrio* species in the hatchery. Out of 347 isolates collected from D-27 to D+15 on nonelective media, 25 isolates (7.2%) were presumed to carry pAQU-MAN, as determined by PCR detection of the *tr* and *catA2* genes. Sequencing of these 25 positive isolates revealed that all pAQU-MAN-carrying bacteria belonged to the Splendidus clade, with *V. splendidus* being the most common (18 isolates) (Fig. 5). Other species included *V. coralliirubri*, *V. crassostreae*, *V. cyclitrophicus*, and *V. lentus*. These results show the rapid and efficient spread of pAQU-MAN between *Vibrio* species in the hatchery.

### pAQU-MAN is a highly stable even in the absence of selective pressure

**Figure 6:**
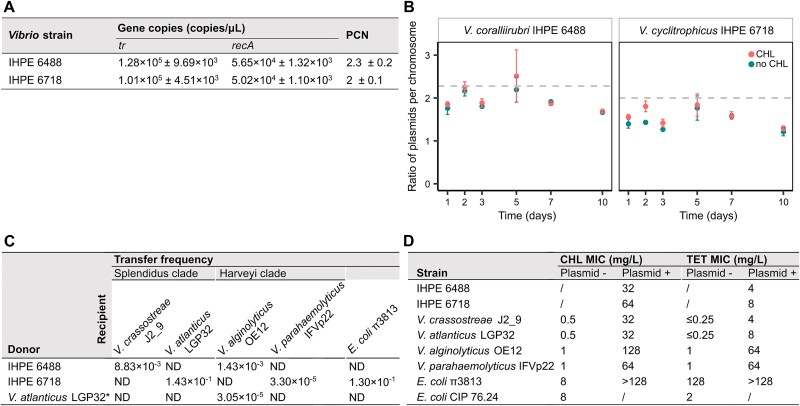
The pAQU-MAN plasmid is a low copy number, stable over time, and capable of conferring phenotypic resistance through conjugative transfer to strains of the *Vibrio harveyi* and Splendidus clades, as well as *E. coli*. (A) pAQU-MAN plasmid is present at two copies per *Vibrio* genome. The plasmid copy number (PCN) for *V. coralliirubri* IHPE 6488 and *V. cyclitrophicus* IHPE 6718 was determined via qPCR by calculating the ratio of the *tr* gene, located on the plasmid, to *recA*, a single-copy chromosomal gene. Values represent the mean ± standard deviation (SD) with *n* = 3. (B) pAQU-MAN plasmid is highly stable. The persistence of the plasmid in *V. coralliirubri* IHPE 6488 and *V. cyclitrophicus* IHPE 6718 was assessed over 10 days, with and without CHL, using the *tr/recA* ratio from the qPCR experiment. Values represent the mean ± standard deviation (SD) with *n* = 4. The gray dashed line represents the previously determined PCN, used as a reference value. (C) High conjugation frequency of the pAQU-MAN plasmid. The frequency of plasmid transfer by conjugation was measured from *Vibrio* strains IHPE 6488 and IHPE 6718 to several strains of the *Vibrio* clades Harveyi and Splendidus, as well as to *E. coli*. The transconjugant strain *V. atlanticus* LGP32^*^, carrying the plasmid, was subsequently used to transfer the plasmid to *V. alginolyticus* OE12, demonstrating the plasmid's ability to self-transfer. ND, not determined. (D) pAQU-MAN confers resistance to CHL and TET. Following conjugation, phenotypic resistance to chloramphenicol (CHL) and tetracycline (TET) conferred by the presence of the plasmid was assessed across a concentration range from 125 to 0.25 mg.l^−1^. The minimum inhibitory concentrations (MICs) of strains carrying pAQU-MAN (Plasmid+) are presented alongside those of strains not carrying the plasmid (Plasmid−). The strain CIP 76.24 was used as a control, following the CLSI and EUCAST standards.

We tested the stability of the pAQU-MAN plasmid in its host strains, in particular *V. coralliirubri* IHPE 6488 and *V. cyclitrophicus* IHPE 6718, by measuring the plasmid-to-genome ratio over a 10-day period with and without selective pressure. The ratio, calculated as the *tr/recA* gene ratio, showed no significant changes between treatments, indicating high plasmid stability. Both strains exhibited stable plasmid copy numbers (2.3 ± 0.2 for IHPE 6488 and 2 ± 0.1 for IHPE 6718) throughout the study (Fig. 6A and B). Therefore, pAQU-MAN also appeared highly stable in its host strains.

### pAQU-MAN is a highly transferable plasmid conferring resistance to CHL and TET

Plasmids that can replicate in a broad range of hosts are of concern as they may drive gene exchange across large phylogenetic distances. Therefore, we tested experimentally the transferability of the pAQU-MAN plasmid using filter conjugation assays. pAQU-MAN was successfully transferred from *V. coralliirubri* IHPE 6488 to *V. crassostreae* J2_9 (i.e. within the Splendidus clade) and *V. alginolyticus* OE12 (i.e. to the Harveyi clade) with conjugation frequencies of 8.83 × 10^−3^ and 1.43 × 10^−3^, respectively. From *V. cyclitrophicus* IHPE 6718, the plasmid was transferred to *V. tasmaniensis* LGP32 (i.e. within the Splendidus clade), *V. parahaemolyticus* IFVp22 (i.e. to the Harveyi clade), and *E. coli* π3813 (outside *Vibrionaceae*) with frequencies of 1.43 × 10^−1^, 3.30 × 10^−5^, and 1.30 × 10^−1^, respectively (Fig. 6C). Plasmid presence in all transconjugants was confirmed by PCR (Fig. S5). We observed that the *V. tasmaniensis* LGP32 transconjugant carrying pAQU-MAN (thereafter labeled with a ^*^) was able to transfer the plasmid to *V. alginolyticus* OE12, demonstrating the potential for further horizontal gene transfer between *Vibrio* clades.

Transconjugants became resistant to CHL and TET, as determined by MIC testing. In the Splendidus clade, the transconjugants J2_9^*^ and LGP32^*^ exhibited MICs of 32 mg.l^−1^ for CHL and 4 and 8 mg.l^−1^ for TET, respectively, compared to wild-type MICs of 0.5 mg.l^−1^ for CHL and ≤0.25 mg.l^−1^ for TET. Similarly, the Harveyi clade transconjugants, OE12^*^ and IFVp22^*^, showed high MICs for CHL (128 and 64 mg.l^−1^) and TET (64 mg.l^−1^) compared to their respective wild-type strains (MIC of 1 mg.l^−1^ for both antibiotics). Finally, the *E. coli* π3813^*^ transconjugant exhibited MICs of 128 mg.l^−1^ for CHL and >128 mg.l^−1^ for TET, whereas the wild-type strain had MICs of 8 and 128 mg.l^−1^, respectively (Fig. 6D). These results confirm that pAQU-MAN confers significant resistance to CHL and TET when transferred between a broad range of bacterial hosts. pAQU-MAN, therefore, has the potential to disseminate antibiotic resistance across large phylogenetic distances.

## Discussion

In a context of global expansion of aquaculture, our study reveals the impact of antibiotic use—here CHL—on the development and dynamics of ARGs, which spread between *Vibrio* populations circulating in hatcheries of *M. gigas* oysters through a highly transmissible plasmid called pAQU-MAN. The conjugative plasmid newly identified in this study was shown to propagate the *catA2* gene, conferring CHL-resistance to a broad range of *Vibrio* species in the hatchery. This plasmid belongs to a conserved family of pAQU-type plasmids, which are found in *Vibrio* spp. and *Photobacterium* spp. Isolated from aquaculture settings worldwide, they are considered emerging multidrug-resistant (MDR) conjugative plasmids in aquatic environments [58]. Initially reported in Japan, followed by studies in China, and more recently on the southeastern Turkish coast of the Black Sea, pAQU-type plasmids could serve as key players in the spread of TET resistance due to the high prevalence of associated ARGs in this plasmid family [58–64]. Our study supports this assumption. Indeed, two copies of *catA2* co-occurred with a *tet(B)* gene in pAQU-MAN. These genes conferred CHL- and TET resistance upon pAQU-MAN conjugative transfer to new bacterial hosts. Moreover, our study showed that the CHL-induced propagation of pAQU-MAN in the hatchery has far-reaching consequences on the spread of unrelated ARG families such as *tet(B),* warning us of the multiple consequences of the use of antibiotics in aquaculture worldwide. This characterization of a pAQU-type plasmid in Europe highlights the ongoing global dissemination of these MDR plasmids, potentially linked to international movement and trade of mollusks and their associated bacteria.

Broad-host-range conjugative plasmids pose a significant risk as they enable the transfer of multiple AMR genes between diverse bacterial species [65]. In this study, we show that pAQU-MAN efficiently transfers to the oyster pathogens *V. crassostreae* and *V. atlanticus* from the Splendidus clade, as well as to *Vibrio* species of the Harveyi clade, which includes human pathogens *V. parahaemolyticus* and *V. alginolyticus.* pAQU-MAN also transfers to *E. coli* at high conjugation efficiency, as also reported for other pAQU-type plasmids [60, 61, 66]. Thus, environmental bacteria carrying pAQU-MAN and other pAQU-type plasmids can disseminate resistance across a wide range of phylogenetically distant host bacteria, including both oyster and human pathogens. pAQU-MAN-mediated CHL-resistance was also found to be host-dependent, with lower resistance levels in commensal *Vibrio* strains and higher resistance in the human pathogens tested. Although pAQU-MAN may not pose an immediate threat to commensal bacteria, its ability to transfer to pathogens and enhance their resistance highlights the need to monitor commensals as reservoirs of pAQU-type plasmids in general. By efficiently spreading across *Vibrionaceae* and beyond, they could facilitate the transfer of diverse ARG families across habitats [3] and increase the risk of AMR transmission to human pathogens, particularly through the consumption of food products such as oysters, which are eaten raw.

We highlighted the existence of a hypervariable region rich in ARGs (up to 23 per plasmid) nested in the highly conserved plasmid backbone of 27 pAQU-type plasmids homologous to pAQU-MAN. This region was also rich in ISs, which are known to play an important role in genome evolution and plasticity [67]. It has recently been demonstrated that ISs are key mediators of ARG exchange between conjugative plasmids and chromosomes [68]; the frequent proximity of ISs with ARGs on plasmids suggests a significant role in the recruitment and transfer of AMR genes between phylogenetically distant bacteria. Comparison of pAQU-type plasmids reveals that they serve as platforms for a broad diversity of ARGs, with a total of 33 different ARGs identified in this study. Although TET resistance determinants such as *tet(59)*, *tet(B)*, *tet(M)* are common in pAQU-type plasmids, *catA2*—present on the newly described pAQU-MAN—was less frequently observed. To our knowledge, only one pAQU-type plasmid, pC704 from *V. alginolyticus*, has been reported to carry the *catA2* gene. pAQU-MAN carries two identical *catA2* sequences within an IR region, whereas pC704 contains a single copy of this gene. IR consists of symmetric nucleotidic sequences with reverse complementary DNA, which are known to promote genome instability [69, 70]. This could further account for the elevated potential for DNA rearrangements in this region, though further research is needed to better understand transposition events within this plasmid and the broader implications for the spread of AMR.

Even though plasmid host range, conjugation efficiency, and plasticity determine the propensity of plasmid-borne ARGs to disseminate efficiently, successful transfer is not guaranteed unless the plasmid is stably maintained in the recipient bacterial host [71]. pAQU-MAN exhibited high stability, demonstrating its ability to persist in a bacterial population without positive selection, as we observed here in monocultures. Like other pAQU-type plasmids, it is a low-copy plasmid, large in size (170 kb), which offers an advantage over high-copy plasmids by reducing the metabolic burden on the host, thereby ensuring higher stability and maintenance [72]. The observed plasmid stability seems to be a common trait of pAQU-type plasmids: pAQU1 plasmid remains stable for up to 30 days in *P. damselae* subsp. *damselae* 04Ya311, without TET [73], while it was maintained in the fly intestine for up to 5 days, their persistence exceeding 7 days in *E. coli* [74]. Attempts to cure the pAQU-MAN plasmid from *V. coralliirubri* IHPE 6488 and *V. cyclitrophycus* IHPE 6718 by introducing a counter-selectable marker (CcdB toxin)—a method we previously used successfully in other *Vibrio* species [75]—were unsuccessful, as we could not obtain plasmid-cured isolates (data not shown). Such difficulties in plasmid loss are often due to the presence of toxin–antitoxin (TA) systems, which assure plasmid maintenance in bacteria and prevent postsegregational killing of plasmid-free cells [76] and are present in >85% of conjugative plasmids [68]. Although we did not identify any canonical TA systems in pAQU-MAN, we did detect sequences with high similarity to putative toxin *rhs* elements (locus tags J47SPOVO_00002, J47SPOVO_00005, J47SPOVO_00008), which share mechanistic similarities with TA modules and may contribute to pAQU-MAN stability [77]. Further investigation is needed to explore the function of these genes and their role in plasmid stability.

Given their highly efficient spread by conjugation and their stability in monocultures, pAQU-MAN plasmids would be predicted to be maintained in the hatchery over long periods of time. However, CHL-resistant strains harboring pAQU-MAN plasmids disappeared from the community after D+15. It should be highlighted that plasmid stability in a monoculture does not necessarily reflect its stability within bacterial communities. Indeed, the community context can affect plasmid spread through a dilution effect, where proficient hosts maintain and repeatedly transfer a plasmid to less proficient hosts—with lower plasmid stability and transmission efficiency—which ultimately drive the plasmid to extinction [3]. Although not investigated here, a variety of bacterial antiplasmid systems have been documented [78–80]. Some have been discovered in the genus *Vibrio,* like the DdmDE system, which rapidly eliminates small, multicopy plasmids from *V. cholerae* [81], or DdmABC, which eliminates large plasmids from the population in the absence of antibiotic selection, by imposing a high fitness cost that favors plasmid-free cells [82]. Although the reason explaining pAQU-MAN decline in the hatchery is not yet resolved, it has been postulated that plasmid defense systems may have contributed to the decline of IncC MDR plasmids in *V. cholerae* seventh pandemic strains [82]. Another alternative is the intervention of predators that target bacteria carrying plasmids. Phages, for example, are known to play a crucial role as bacterial predators, shaping the structure, function, and evolution of natural microbial communities [83]. In previous studies, we observed that *Vibrio* populations are highly dynamic in natural ecosystems [75], likely in part due to phage predation [84]. Although most phages rely on chromosomally encoded cell surface structures as receptors, plasmid-dependent phages have been identified that utilize plasmid-encoded conjugation proteins or pilus [85]. These plasmid-dependent phages have been proposed to serve as a significant but often overlooked constraint on the spread of conjugative plasmids [86].

## Conclusion

This study represents the earliest documented characterization of pAQU-type MDR plasmids in Europe, previously limited to Asia and Turkey, revealing a recent concerning spread with implications for aquaculture management. Although banned in the European Union (EU) and many other countries, CHL use can persist sporadically in key shellfish aquaculture regions [9]. We showed here that CHL promotes the selection of plasmid-borne ARGs, specifically *catA2* and *tet(B),* driving MDR. The genetic localization of ARGs within IS-rich regions suggests that DNA exchanges likely occur upon environmental selection, further facilitating resistance selection and spread. The high stability of the pAQU-MAN plasmid in *Vibrio* bacteria, even in the absence of selective pressure, exacerbates the potential for resistance dissemination and likely explains the persistence of these genes in the hatchery over several weeks. However, the natural decline of *catA2* prevalence, becoming undetectable in oyster progenies by 3 months, when they are ready for deployment in the field, is reassuring as it suggests that resistant bacteria are unlikely to spread through oysters into the environment. Nevertheless, the release of CHL-resistant (*catA2*-positive) bacteria during broodstock conditioning should be considered in treating aquaculture facility wastewater. Although initially found in commensal *Vibrio* isolates, the pAQU-MAN plasmid can easily transfer to pathogenic oyster and human *Vibrio* strains, and to *E. coli*, posing significant risks to both animal and human health. Overall, this study highlights the critical need for surveillance of *Vibrio* strains carrying pAQU-type plasmids and the establishment of standardized guidelines for detecting resistance in aquatic environments. Such measures are essential for improving the management and containment of AMR in aquatic systems, safeguarding both human and animal health, and providing valuable insights into future strategies to mitigate AMR risks.

## Supplementary Material

Supplementary_materials_MOUGIN_ISME_wraf163

## Data Availability

The datasets generated and analyzed during the current study are available in the European Nucleotide Archive (ENA) repository. Whole-genome raw data of antibiotic-resistant bacteria were deposited under ENA accession number PRJEB89088 (ERX14309669 to ERX14309695), and are available on MicrosScope platform MaGe (“Magnifying Genomes,” https://mage.genoscope.cns.fr). Raw data of pool sequencing of antibiotic-resistant bacteria is available under ENA accession number PRJEB89075 (ERX14309344 to ERX14309355). Details of experiment accession numbers are provided in Tables S6 and S12 of the Supplementary Data.
