# Supplementary material for: Antibiotic use in oyster hatcheries promotes rapid spread of a highly transferable and modular resistance plasmid in Vibrio
Source: ISME J. 2025 Aug 13;19(1):wraf163. doi: 10.1093/ismejo/wraf163 (PMC12406693; doi:10.1093/ismejo/wraf163)
Supplement: Supplementary_materials_MOUGIN_ISME_wraf163 [file supplementary_materials_mougin_isme_wraf163.pdf]

Supplementary

Antibiotic use in oyster hatcheries promotes rapid spread of a highly transferable and modular resistance plasmid in *Vibrio*

Julia Mougin[1], Yannick Labreuche[1], Viviane Boulo[1], David Goudenège[2], Jamal Saad[1], Gaelle Courtay[1], Jacqueline Le Grand[3,4], Oriane Chevalier[1], Juliette Pouzadoux[1], Caroline Montagnani[1], Marie-Agnès Travers[1], Bruno Petton[3,4], Delphine Destoumieux-Garzón[1*]

1 IHPE, Université de Montpellier, CNRS, IFREMER, Université de Perpignan Via Domitia, place E. Bataillon, 34090 Montpellier, France

2 IFREMER, IRSI, SeBiMER Service de Bioinformatique de l'Ifremer, ZI de la Pointe du Diable, 29280 Plouzané, France

3 IFREMER, Unité écoPHYsiologie et Traits d'histoire de vie des orgaNismES marinS (PHYTNESS), ZI de la Pointe du Diable, 29280 Plouzané, France

4 Laboratoire des sciences de l'environnement marin (LEMAR), Université de Bretagne Occidentale, CNRS, Institut de recherche pour le développement (IRD), Ifremer, Technopôle Brest-Iroise, Rue Dumont D'urville, 29280 Plouzané, France

1. Supplementary Material and Methods

1.1. Oyster rearing: phases and maintenance

Juvenile production of *Crassostrea gigas* comprised two distinct periods. The Period 1, lasting 47 days, involved broodstock conditioning required for gametogenesis, culminating in reproduction. The Period 2, lasting 105 days, involved the rearing of embryos up to the spat stage. The initial 41 days of Period 2, focusing on rearing embryos up to the young spat stage (2 mm in size), were conducted in Argenton, Finistère, France. The remaining days were spent rearing the young spat in Bouin, Vendée, France, where they reached a size of 2 to 4 cm by the end of the rearing period.

During Period 1 and 2, the rearing tanks were supplied with seawater, salinity 34g $^{l-1}$, filtered at 10 microns and specifically at 1 micron for the embryo and larvae rearing phase (D0-D+15), before undergoing ultraviolet irradiation. The same diet was used to feed broodstock and larvae, consisting of a biomass-equivalent mixture of *Isochrysisaffinis galbana* (CCAP 927/14 also named T-Iso) and *Chaetoceros gracilis* (UTEX LB2658). Spat grow-out was provided by *Skeletonema sp.* Phytoplankton distribution was continuous and automated (peristaltic pumps) in the flow of seawater feeding the rearing structures. Single-cell algae were produced in successive batches (300L). The phytoplanktonic environment of spawners, larvae and spat was maintained at 1500 µm^3 µl^{-1}. It was quantified in the effluent leaving the rearing tanks using a particle counter (Multisizer 3, Beckman coulter) equipped with a 100 µm aperture.

Period 1: Broodstock conditioning and reproduction

Since 2011, a batch of 1,500 wild oyster spat has been collected each year in Fouras (Marennes-Oléron, France; 46° 0' 43" N, 1° 7' 3" W) and transferred to Ifremer Aber-Benoît (northern Brittany, France, 48° 34' 29.976" N, 4° 36' 18.378" W). On January 12th 2021, 166 adult oysters were transferred to the Ifremer experimental facility in Argenton (France) for the gametogenesis period. The oysters were cleaned of their epibionts by brushing and rinsing in fresh water before being placed in 4 tanks (2 tanks per

antibiotic broodstock treatment). Each of the 4 400 L tanks contained 44 oysters from the 8 age classes (4 individuals born in 2011, 4 in 2012, 4 in 2013, 4 in 2014, 5 in 2015, 5 in 2016, 8 in 2017 and 10 in 2018). The average total biomass per tank was 5277 g for an average individual weight around 120 g. Among the 4 tanks, 2 of them were not treated with antibiotic (A-), while broodstock within the 2 remaining tanks were treated with Chloramphenicol (CHL) for 6 days (3 repetitive baths of 48 h with 8 $mg.L^{-1}$ of CHL in seawater), as described previously [1,2]. Details regarding antibiotic usage are provided in Table S1. To ensure antibiotic removal at the water outlet, filtration through an activated carbon filter was performed. Upon arrival, CHL was administered immediately to the oysters without any feeding. The 6-day antibiotic treatment concluded just as feeding commenced, with the oysters being kept in seawater maintained at a constant temperature of 17°C throughout the subsequent conditioning period.

Table S1: Antibiotic usage.

Veterinary drug	Antibiotic molecule	Abbreviation	Concentration in treated tanks	Animal	Time
CHLORAMPHENICOL	Chloramphenicol	CHL	8 $mg.L^{-1}$	Broodstock	6 days
FLUMISOL® 36%	Flumequine 360 mg/mL	FLU	0.026 $mL.L^{-1}$ or 9.4 $mg.L^{-1}$ of FLU	Larvae	4 days
NUFLOR® 300mg/mL	Florfenicol 300mg/mL	FLO	0.022 $mL.L^{-1}$ or 6.6 $mg.L^{-1}$ of FLO	Larvae	4 days

On March 8th 2021, after 47 days of conditioning, gametes from mature male and female oysters of each of the 4 tanks were obtained separately by lacerating the gonads (i.e. two reproductions per experimental condition; control condition A- and CHL treated broodstock). The genital products of the broodstock from each tank were pooled into two 5-L volumes, with 50 oocytes per ml and 3 to 5 spermatozoa per oocyte, over a period of 2 hours, which was necessary for oocyte fertilization. The embryos were then placed for 48 hours in eight 140-L tanks at a density of 100 embryos per milliliter of seawater at 21°C, without water renewal or food supply, and with aeration (two tanks per clutch, i.e. four tanks per antibiotic broodstock treatment). As such, each individual broodstock tank produced two embryo tanks, resulting in a total of 4 tanks per antibiotic broodstock treatment and 8 tanks overall. At the end of the 48-hour embryogenesis period, the survival of D-larvae was estimated.

Period 2: Larval rearing phase

On March 10th 2021, at day 2 (D+2) of embryonic development, the D-larvae were distributed volumetrically in 12 tanks of 140-L to achieve a density of 10 $^{ML-1}$ larvae (1,400,000 larvae per tank; 4 tanks per antibiotic larvae treatment). A number of 4 larvae tanks were not treated with antibiotics: 2 tanks originating from A- broodstock treatment (A-/A-) and 2 from CHL broodstock treatment (CHL/A-). Additionally, 4 larvae tanks received flumequine (FLU) for 4 days: 2 tanks from A- broodstock (A-/FLU) and 2 from CHL broodstock (CHL/FLU). Lastly, 4 other tanks were treated with florfenicol (FLO) for 4 days: 2 tanks from A- broodstock (A-/FLO) and 2 from CHL broodstock (CHL/FLO). Larvae underwent 2 repetitive baths of 48 h of antibiotics with FLU at 9.4 $mg.L^{-1}$ and FLO at 6.6 $mg.L^{-1}$ in seawater, as per veterinary recommendations. The two treatments were carried out between day 2 (feeding start date) and day 6 of larval rearing at a temperature between 22°C (D2) and 25°C (D+6). Details of antibiotic usage are provided in Table 1.

Rearing tanks were cleaned and seawater renewed on days 4, 7, 9, 11, 13, 14 and 15 of rearing. On day 11, the larval density was lowered to 3^{ML-1} larvae in all tanks without sorting (corresponding to a fraction of the population to ensure optimal development of the larvae kept in rearing). The seawater temperature was gradually increased from 22°C (D+2) to 25°C (D+6) and then maintained at this value until the end of the larval rearing phase. Larvae in conditions A-/A-, A-/FLU, CHL/A-, CHL/FLU and CHL/FLO were ready for pediveliger stage by day 15, and those in condition A-/FLO by day 17. Metamorphosis took place on shellfish microcracks (1 micron) in a specific structure (100,000 larvae from each tank for as many baskets, i.e. two baskets per condition). The shatter was removed on day 28 by sieving (400-micron mesh sieve) and metamorphosis was assessed on day 30 (April 8th 2021). After this date, rearing continued until day 41 (April 19th 2021, size >1.4 mm), when the young spats were transferred to the

Ifremer facility in Bouin, which is better suited for the rearing of larger-sized spat. The seawater temperature was gradually lowered from 25°C to 20°C between days 30 and 41 to reach the seawater temperature of the new structure (uncontrolled temperature).

1.2. Sampling of water and oyster tissues

Water samples
An amount of 100 µL water samples were collected from the tanks, during the broodstock conditioning phase (Period 1), on days 20, 34, and 47, and during the larval rearing phase (Period 2), on days 2, 7, and 15. A minimum of two samples were taken from the two-replicate tanks, (n=4 for each condition). Pure or 1/10 diluted sample were then plated on selective Thiosulfate-Citrate-Bile-Saccharose (TCBS) media to collect bacterial isolates. Additional water samples were collected for water quality control, from the tank near the water inlet and plated on selective media to verify that the water was not a source of antibiotic-resistant bacteria.

Oyster gametes and broodstock
At the end of the broodstock conditioning phase (Period 1), on day 0 (Fig. 1 D0), oyster gametes were collected from reproduction experiment. Samples consisted of 4000 oocytes or 1 mL of sperm, were homogenized in 2 mL of sterile seawater using an Ultra-Turrax T25 for 5 seconds (IKA®-Labortechnik, Janke & Kunkel). For each gamete type, two samples per tanks were collected (n=4 for each condition). An amount of 100 µL of sample were then plated on selective media to collect bacterial isolates, as described in 3.3. A number of 8 adult oysters from each of the two-replicate tanks (n=16 for each condition), were collected on day 0 as well, immediately frozen in liquid nitrogen and conserved at -80 °C until further analysis.

Larval samples
During the larval rearing phase (Period 2), larvae were collected on day 2 just before administering the antibiotic. Post-treatment, larvae samples were also collected on days 7 and 15 (Fig. 1 D+7 and D+15). Each larval sample consisted of 5000 larvae, rinsed with sterile seawater using a 40-micron sieve, and homogenized in 2 mL of sterile seawater with an Ultra-Turrax T25 for 5 seconds (IKA®-Labortechnik, Janke & Kunkel). An amount of 100 µL of sample were then plated on selective media to collect bacterial isolates, while 4 larval sample per tanks (n=8 for each condition) were immediately frozen in liquid nitrogen and conserved at -80 °C until further analysis.

Spat samples
On day 105 of spat rearing phase (Period 2), only spat samples were collected and processed as previously described, as on day 15 no bacteria were isolated from water on selective media containing antibiotics. Each spat sample consisted of three individuals, homogenized in 10 mL of sterile seawater using an Ultra-Turrax T25 for 2 × 10 seconds (IKA®-Labortechnik, Janke & Kunkel), (n=3 for each condition). An amount of 100 µL of sample were then plated on selective media to collect bacterial isolates, while some samples (n=6 for each condition) were immediately frozen in liquid nitrogen and conserved at -80 °C until further analysis.

1.3. Colony-PCR conditions for plasmid conjugation confirmation

To confirm that the transconjugants were true recipient derivatives and not spontaneous chloramphenicol (CHL)-resistant mutants, both strain and plasmid verification were performed using colony-PCR. A single colony was picked from the agar plates and resuspended in 100 µL of Milli-Q water. The suspension was then boiled at 99°C for 10 minutes to lyse the cells. The resulting lysate was diluted 1:100 in DNAse free water to minimize the presence of PCR inhibitors. The PCR reaction mixture was prepared using the previously described primers as follows: 5 µL of 5× PCR reaction buffer, 0.5 µL of 10 mM dNTP, 0.25 µL of each primer (stock at 100 µM), 0.2 µL of GoTaq® DNA Polymerase

(Promega), 1 µL of boiled bacteria, and 15.3 µL of nuclease-free water to a final volume of 25 µL. The PCR reaction was run on a Thermal Cycler (Eppendorf), under the following conditions: 2 min at 95 °C, followed by 30 cycles of 30 s at 95 °C, 30 min at 55 °C, and 30 s at 72 °C. The final cycle was followed by an additional 3 min of extension at 72 °C. All PCR experiments contained a positive control, a negative control, and a no template control (NTC), DNA-free. The size of the PCR product was verified by ethidium bromide agarose gel electrophoresis (1%).

Table S2. Primers used in this study

Target gene	Detection	Primer name	Primer sequence (5'-3')	Amplicon length (bp)	Efficiency (%) for qPCR	R^2 for qPCR	Reference
Universal 16S rRNA	Bacteria	Total_B_926F<br>Total_B_1062R	AAACTCAAAKGAATTGACGG<br>CTCACRRCACGAGCTGAC	136	96.2	0.997	[3]
catA2	Chloramphenicol acetyltransferase 2	CMLA R2-3<br>CMLA F3	CGCGGTTATCTTCCTGCTGA<br>CCTGGGTGAGTTTTGACGGA	101	100.0	0.999	This study
		catBv6 R1-F2	ACCAATTACCGTGTCCCCTG				
tr1	Transposase gene 1 from R-Plasmid for PCR	240624-1<br>240624-2	GTATCGATAAGCTTGATATCGAATTCGCGTGGATTACTATGGTGCC<br>CCCCCGGGCTGCAGGAATTCGCGATCCAGACTGGCATCAC	545	/	/	This study
tr2	Transposase gene 2 from R-Plasmid for qPCR	230724-1<br>230724-2	CGAGCTGTGGAATGAATGCG<br>TGCTCGCTTAATAGCCTCCG	133	104.0	0.989	This study
recA	*C*hromosomes of J7R_L_CmR_R01 and J47R_L_CmR_R01 (single copy gene)	230724-3<br>230724-4	TAGCCGCAGCCCTTGGTCAG<br>GGCCACCAGCACCTAGTGCG	138	111.0	0.988	This study
thrB	*V. crassostreae* strain J2-9	160724-1<br>160724-2	AGGATGTGATTGCCCATGGC<br>CCAGTGCTCCAGCTGAAGTG	166	/	/	This study
lpp Putative outer membrane lipoprotein 1808171	*V. atlanticus* strain LGP32	160724-3<br>160724-4	TCGGCTGCTGATGACCGAAC<br>TTCCCCAACCATTTCCTAGC	331	/	/	This study
CuAO Copper amine oxidase	*V. alginolyticus* strain OE12	270524-1<br>270524-2	GTATCGATAAGCTTGATATCGAATTCATTGGTCGTTGCCTTCGTGG<br>CACTACTATTGAGGTGCAGATTCCACTTCTTTTTGAGGGTATTATG	500	/	/	This study
Putative type III secretion system apparatus protein VscR2	*V. parahaemolyticus* IFVp22	IFVp22R<br>IFVp22F	TAAGCCCGGCCTTGAGCGTG<br>TAAGCCCGGCCTTGAGCGTG	394	/	/	This study
uspA	*E. coli*	uspAR<br>uspAF	CCGATACGCTGCCAATCAGT<br>ACGCAGACCGTAGGCCAGAT	884	/	/	[4]

Table S3: Metadata of published R-plasmids used for comparison (nd: not determined)

Species	Strain	Plasmid	Length (pb)	Accession	GI	PMID	Isolation source/host	Isolation location	Isolation year	Submission date	Sequencing technology	GC %	tRNAs	ncRNAs	CDSs	pseudo	sORFs	gaps
Vibrio sp.	04Ya090	pAQU2	160406	AB856327.1	577007824	24860553	sediment from coastal aquaculture site	Japan: Kagawa	nd	21-MAR-2022	GS FLX	42.6	0	4	191	2	1	1
Vibrio penaeicida	TUMSAT-OK1	pTUMSAT-OK1	150136	AP025154.1	2091883084	unpublished	nd	nd	nd	09-SEP-2021	Illumina / Nanopore	43.3	0	0	187	1	0	0
Vibrio penaeicida	TUMSAT-OK2	pTUMSAT-OK2	150127	AP025157.1	2091883087	unpublished	nd	nd	nd	10-SEP-2021	Illumina / Nanopore	43.3	0	0	189	2	0	0
Photobacterium damselae	04Ya311	pAQU1	203929	AP026782.1	2302523932	38078722	coastal sea water	Japan: Kagawa	2004	19-MAR-2024	Illumina / Nanopore	43.2	0	5	225	1	1	0
Vibrio alginolyticus	FDAARGOS_108	CP014052.1	217123	CP014052.1	1345487382	unpublished	seawater	Puerto Rico	nd	30-SEP-2019	Illumina / PacBio	43.4	0	0	255	2	0	0
Vibrio harveyi	345	p345-185	185327	CP025539.1	1318793482	unpublished	*epinephelus oanceolutus*	China: Shenzhen	2013	19-APR-2018	Illumina / PacBio	43.3	0	3	214	1	1	0
Vibrio owensii	20160513VC2W	p2	186279	CP030801.1	1877708918	unpublished	shrimp	China	2016	22-JUL-2020	PacBio	42.7	0	3	215	2	1	0
Vibrio parahaemolyticus	20160303005-1	pVPSD2016-2	193123	CP034301.1	1797010228	unpublished	*litopenaeus vannamei*	China: Weifang	2016	15-JAN-2020	PacBio	43.7	0	1	206	0	0	0
Vibrio parahaemolyticus	2012AW-0154	CP035701.1*	166671	CP035701.1	1776325518	unpublished	nd	nd	nd	18-NOV-2019	PacBio	42.9	0	0	202	0	0	0
Vibrio parahaemolyticus	NUK/7	p_2743	156346	CP066163.1	1948800598	unpublished	shrimp	India	2017	21-DEC-2020	Illumina	43.2	0	0	195	0	0	1
Vibrio parahaemolyticus	Vp2015094	Vp2015094_p1	191858	CP080480.1	2440514261	35660661	shellfish	China: Liaoning Province	2015	06-FEB-2023	PacBio	43.2	0	5	217	1	1	0
Vibrio furnissii	MT14	pMT14	207270	CP115190.1	2508600654	unpublished	*ruditapes philippinarum*	China: Nanjing	2021	22-MAY-2023	Illumina / Nanopore	43.3	0	0	223	1	0	0
Vibrio sp.	YMD68	CP124615.1	168227	CP124615.1	2501483974	unpublished	sediment	China: The Yellow Sea	2022	09-MAY-2023	Illumina / Nanopore	43.1	0	0	208	0	0	0
Vibrio harveyi	SB1	SB1_p1	153407	CP125877.1	2508231654	unpublished	*lates calcarifer*	India: Chennai	2020	22-MAY-2023	Illumina / PacBio	43.2	0	0	190	0	0	0
Vibrio alfacsensis	VA-1	plas2	221646	CP140105.1	2636768650	unpublished	*scophthalmus maximus*	China: Weihai	2021	18-DEC-2023	Illumina / Nanopore	43.1	0	5	240	1	1	0

Species	Strain	Plasmid	Length (pb)	Accession	GI	PMID	Isolation source/host	Isolation location	Isolation year	Submission date	Sequencing technology	GC %	tRNAs	ncRNAs	CDSs	pseudo	sORFs	gaps
Vibrio parahaemolyticus	vp-201806	pA-vp-201806	157684	CP150859.1	2733735094	unpublished	*penaeus vannamei*	China: Fujian	2018	17-MAY-2024	PacBio	43.7	0	0	178	1	0	0
Vibrio parahaemolyticus	NJIFDCVp7	pVP7-1	229659	CP150866.1	2733740091	unpublished	seafood	China: Nanjing	2021	17-MAY-2024	Illumina / Nanopore	44.1	0	1	232	1	0	0
Vibrio parahaemolyticus	V36	pVPH1	183730	KP688397.1	760459642	25779581	shrimp	Hong Kong	nd	06-APR-2020	Illumina	45.2	0	0	212	1	0	0
Vibrio parahaemolyticus	2011VPH2	pVPH2	198487	KP791968.1	808352731	unpublished	nd	Hong Kong	nd	06-APR-2020	Illumina	44.7	0	1	244	2	1	1
Vibrio alginolyticus	nd	pVAS19	187130	KX957968.1	1131738249	unpublished	shrimp	China	2015	06-APR-2020	Illumina / PacBio	44.5	0	2	215	0	1	0
Vibrio alginolyticus	nd	pVAS114	206274	KX957969.1	1131738465	unpublished	shrimp	China	2015	06-APR-2020	Illumina / PacBio	45.3	1	0	226	1	0	0
Vibrio parahaemolyticus	nd	pVPS43	194479	KX957970.1	1131738691	unpublished	shrimp	China	2013	06-APR-2020	Illumina / PacBio	45.5	1	0	214	1	0	0
Vibrio parahaemolyticus	nd	pVPS62	184719	KX957971.1	1131738909	unpublished	chicken	China	2015	06-APR-2020	Illumina / PacBio	43.3	0	2	212	1	1	0
Vibrio parahaemolyticus	nd	pVPS91	163005	KX957972.1	1131739122	unpublished	shrimp	China	2015	06-APR-2020	Illumina / PacBio	43.4	0	0	204	0	0	0
Vibrio alginolyticus	C1579	pC1579	236774	MN865127.1	1840379910	unpublished	shrimp	China	2016	16-MAY-2020	Illumina / PacBio	44.5	0	0	240	0	0	0
Vibrio alginolyticus	704	pC704	193433	OP958859.1	2520264154	unpublished	shrimp	China	nd	21-JUN-2023	Illumina	44.2	1	3	222	0	0	0
Vibrio parahaemolyticus	Vb0677	pVb677-tet	183307	OQ622008.1	2552394410	unpublished	shrimp	China	2015	30-JUL-2023	Illumina / PacBio	42.8	0	3	213	1	1	0

Table S4. Details of strains used for plasmid conjugation.

Name	Species	Year	Place	Sample	Reference
J2-9	*V. crassostreae*	2011	Baybof Brest, Pointe du Château	Oysters, *Crassostrea gigas*	[5]
LGP32	*V. atlanticus*	2001	French Atlantic Coast	Oysters suffering summer mortalities, *Crassostrea gigas*	[6,7]
IFVp22	*V. parahaemolyticus*	1999	France (English Channel)	Mussel, *Mytilus edulis*	[8]
OE12	*V. alginolyticus*	2021	Thau Lagoon, France	Oysters, *Crassostrea gigas*	Not published, IHPE laboratory, Montpellier
π3813	*E. coli*	Laboratory donor strain frequently used for plasmid conjugation.			[9]

2. Supplementary results

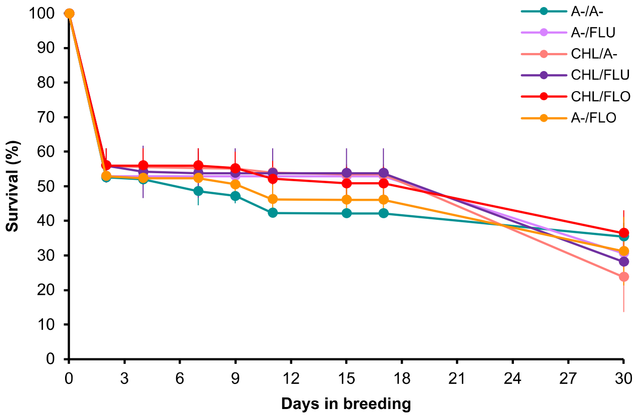

Figure S1. Antibiotic have limited impact on oyster survival. Survival is calculated for progenies from the six antibiotic treatment conditions (A-/A-, A-/FLU, CHL/A-, CHL/FLU, CHL/FLO, A-/FLO) from D0 to D30. Mean ± standard deviation is represented. Within the legend's name, the first part denotes the broodstock treatment (A- or CHL), while the second denotes the larvae treatment (A-, FLU, or FLO). Only one animal died during broodstock conditioning, in CHL-treated oysters. Gonadal development was robust, facilitating effective reproduction. Fertilization rates exceeded 94% 1.5 h post-fertilization, regardless of treatment. At 48 h post-fertilization, D-larvae (D+2) survival was similar between untreated (55.9%) and antibiotic-treated (52.7%) groups. No significant differences in larval mortality were observed across treatments, except at D+15. At this time point, larval survival in the antibiotic-free group (A-/A-) was lower, reaching 74.1%, while antibiotic-treated groups exhibited survival rates above 97%. However, by D+30 of rearing, survival rates among metamorphosed juvenile oysters were not significantly different across the six tested conditions.

Table S5: Bacterial counting on TCBS and TCBS+10µg/mL of chloramphenicol (CHL). * For seawater samples, data are expressed as CFU/ml, for gametes data are expressed as CFU/mL of sperm (no bacteria were isolated from oocytes), for larvae samples data are expressed as CFU/1000 larvae, while for spat samples data are expressed as CFU/g.

Sample	Genitor tank number or pool	Sampling number	Hatchery time	Broodstock antibiotic treatment history	Larvae antibiotic treatment history	Culture condition	Mean CFU/sample*
Seawater	B3	91bis	D+20	A-	/	TCBS	2550,0
Seawater	B8	94bis	D+20	A-	/	TCBS	1100,0
Seawater	B3	91bis	D+20	A-	/	TCBS+10µg/mL CHL	10,0
Seawater	B8	94bis	D+20	A-	/	TCBS+10µg/mL CHL	20,0
Seawater	B1	89 bis	D+20	CHL	/	TCBS	1100,0
Seawater	B7	93bis	D+20	CHL	/	TCBS	2260,0
Seawater	B1	89 bis	D+20	CHL	/	TCBS+10µg/mL CHL	1240,0
Seawater	B7	93bis	D+20	CHL	/	TCBS+10µg/mL CHL	530,0
Seawater	B3	115	D+34	A-	/	TCBS	60,0
Seawater	B8	118	D+34	A-	/	TCBS	190,0
Seawater	B3	115	D+34	A-	/	TCBS+10µg/mL CHL	0,0
Seawater	B8	118	D+34	A-	/	TCBS+10µg/mL CHL	0,0
Seawater	B1	113	D+34	CHL	/	TCBS	40,0
Seawater	B7	117	D+34	CHL	/	TCBS	110,0
Seawater	B1	113	D+34	CHL	/	TCBS+10µg/mL CHL	250,0
Seawater	B7	117	D+34	CHL	/	TCBS+10µg/mL CHL	40,0
Seawater	B3	123	D0	A-	/	TCBS	286,7
Seawater	B8	126	D0	A-	/	TCBS	170,0
Seawater	B3	123	D0	A-	/	TCBS+10µg/mL CHL	0,0
Seawater	B8	126	D0	A-	/	TCBS+10µg/mL CHL	0,0

Sample	Genitor tank number or pool	Sampling number	Hatchery time	Broodstock antibiotic treatment history	Larvae antibiotic treatment history	Culture condition	Mean CFU/sample*
Seawater	B1	121	D0	CHL	/	TCBS	46,7
Seawater	B7	125	D0	CHL	/	TCBS	150,0
Seawater	B1	121	D0	CHL	/	TCBS+10µg/mL CHL	6,7
Seawater	B7	125	D0	CHL	/	TCBS+10µg/mL CHL	23,3
Seawater	B3	144	D+2	A-	/	TCBS	5960,0
Seawater	B3'	145	D+2	A-	/	TCBS	5880,0
Seawater	B8	150	D+2	A-	/	TCBS	10000,0
Seawater	B8'	151	D+2	A-	/	TCBS	10000,0
Seawater	B3	144	D+2	A-	/	TCBS+10µg/mL CHL	1000,0
Seawater	B3'	145	D+2	A-	/	TCBS+10µg/mL CHL	1000,0
Seawater	B8	150	D+2	A-	/	TCBS+10µg/mL CHL	1590,0
Seawater	B8'	151	D+2	A-	/	TCBS+10µg/mL CHL	660,0
Seawater	B1	140	D+2	CHL	/	TCBS	660,0
Seawater	B1'	141	D+2	CHL	/	TCBS	2840,0
Seawater	B7	148	D+2	CHL	/	TCBS	5080,0
Seawater	B7'	149	D+2	CHL	/	TCBS	6720,0
Seawater	B1	140	D+2	CHL	/	TCBS+10µg/mL CHL	20,0
Seawater	B1'	141	D+2	CHL	/	TCBS+10µg/mL CHL	80,0
Seawater	B7	148	D+2	CHL	/	TCBS+10µg/mL CHL	530,0
Seawater	B7'	149	D+2	CHL	/	TCBS+10µg/mL CHL	64,0
Gametes	B3	135	D0	A-	/	TCBS	135,0
Gametes	B8	139	D0	A-	/	TCBS	25,0
Gametes	B3	135	D0	A-	/	TCBS+10µg/mL CHL	0,0
Gametes	B8	139	D0	A-	/	TCBS+10µg/mL CHL	0,0
Gametes	B1	129	D0	CHL	/	TCBS	150,0
Gametes	B7	137	D0	CHL	/	TCBS	95,0
Gametes	B1	129	D0	CHL	/	TCBS+10µg/mL CHL	30,0
Gametes	B7	137	D0	CHL	/	TCBS+10µg/mL CHL	0,0
Larvae	B3	156	D+2	A-	A-	TCBS	190,0
Larvae	B3'	157	D+2	A-	A-	TCBS	243,0
Larvae	B8	162	D+2	A-	A-	TCBS	1200,0
Larvae	B8'	163	D+2	A-	A-	TCBS	1040,0
Larvae	B3	156	D+2	A-	A-	TCBS+10µg/mL CHL	0,0
Larvae	B3'	157	D+2	A-	A-	TCBS+10µg/mL CHL	0,0
Larvae	B8	162	D+2	A-	A-	TCBS+10µg/mL CHL	216,0
Larvae	B8'	163	D+2	A-	A-	TCBS+10µg/mL CHL	41,0
Larvae	B1	152	D+2	CHL	A-	TCBS	85,0
Larvae	B1'	153	D+2	CHL	A-	TCBS	217,0
Larvae	B7	160	D+2	CHL	A-	TCBS	606,0
Larvae	B7'	161	D+2	CHL	A-	TCBS	330,0
Larvae	B1	152	D+2	CHL	A-	TCBS+10µg/mL CHL	11,0
Larvae	B1'	153	D+2	CHL	A-	TCBS+10µg/mL CHL	1,0
Larvae	B7	160	D+2	CHL	A-	TCBS+10µg/mL CHL	8,0
Larvae	B7'	161	D+2	CHL	A-	TCBS+10µg/mL CHL	12,0
Larvae	B3	177	D+7	A-	A-	TCBS	5298,4
Larvae	B8	168	D+7	A-	A-	TCBS	4615,4
Larvae	B3	177	D+7	A-	A-	TCBS+10µg/mL CHL	0,0
Larvae	B8	168	D+7	A-	A-	TCBS+10µg/mL CHL	0,0
Larvae	B3	178	D+7	A-	FLU	TCBS	119,0
Larvae	B8	169	D+7	A-	FLU	TCBS	4483,9
Larvae	B3	178	D+7	A-	FLU	TCBS+10µg/mL CHL	0,0
Larvae	B8	169	D+7	A-	FLU	TCBS+10µg/mL CHL	16,1
Larvae	B3	179	D+7	A-	FLO	TCBS	0,0
Larvae	B8	170	D+7	A-	FLO	TCBS	0,0
Larvae	B3	179	D+7	A-	FLO	TCBS+10µg/mL CHL	0,0
Larvae	B8	170	D+7	A-	FLO	TCBS+10µg/mL CHL	0,0
Larvae	B1	174	D+7	CHL	A-	TCBS	1317,3
Larvae	B1	174	D+7	CHL	A-	TCBS+10µg/mL CHL	86,5
Larvae	B7	171	D+7	CHL	A-	TCBS	15,8
Larvae	B7	171	D+7	CHL	A-	TCBS+10µg/mL CHL	0,0
Larvae	B1	175	D+7	CHL	FLU	TCBS	0,0
Larvae	B7	172	D+7	CHL	FLU	TCBS	5333,3
Larvae	B1	175	D+7	CHL	FLU	TCBS+10µg/mL CHL	0,0
Larvae	B7	172	D+7	CHL	FLU	TCBS+10µg/mL CHL	66,7
Larvae	B1	176	D+7	CHL	FLO	TCBS	0,0
Larvae	B7	173	D+7	CHL	FLO	TCBS	0,0
Larvae	B1	176	D+7	CHL	FLO	TCBS+10µg/mL CHL	0,0
Larvae	B7	173	D+7	CHL	FLO	TCBS+10µg/mL CHL	0,0

Sample	Genitor tank number or pool	Sampling number	Hatchery time	Broodstock antibiotic treatment history	Larvae antibiotic treatment history	Culture condition	Mean CFU/sample*
Larvae	B3	226	D+15	A-	A-	TCBS	621,2
Larvae	B8	224	D+15	A-	A-	TCBS	0,0
Larvae	B3	226	D+15	A-	A-	TCBS+10µg/mL CHL	0,0
Larvae	B8	224	D+15	A-	A-	TCBS+10µg/mL CHL	0,0
Larvae	B3	228	D+15	A-	FLU	TCBS	117,6
Larvae	B8	225	D+15	A-	FLU	TCBS	18,2
Larvae	B3	228	D+15	A-	FLU	TCBS+10µg/mL CHL	0,0
Larvae	B8	225	D+15	A-	FLU	TCBS+10µg/mL CHL	0,0
Larvae	B3	230	D+15	A-	FLU	TCBS	0,0
Larvae	B8	233	D+15	A-	FLU	TCBS	0,0
Larvae	B3	230	D+15	A-	FLU	TCBS+10µg/mL CHL	0,0
Larvae	B8	233	D+15	A-	FLU	TCBS+10µg/mL CHL	0,0
Larvae	B3	219	D+15	A-	FLO	TCBS	13,3
Larvae	B8	221	D+15	A-	FLO	TCBS	7,1
Larvae	B3	219	D+15	A-	FLO	TCBS+10µg/mL CHL	0,0
Larvae	B8	221	D+15	A-	FLO	TCBS+10µg/mL CHL	0,0
Larvae	B1	222	D+15	CHL	A-	TCBS	3921,2
Larvae	B7	216	D+15	CHL	A-	TCBS	156,5
Larvae	B1	222	D+15	CHL	A-	TCBS+10µg/mL CHL	422,1
Larvae	B7	216	D+15	CHL	A-	TCBS+10µg/mL CHL	0,0
Larvae	B1	227	D+15	CHL	FLU	TCBS	138,2
Larvae	B7	229	D+15	CHL	FLU	TCBS	48,3
Larvae	B1	227	D+15	CHL	FLU	TCBS+10µg/mL CHL	0,0
Larvae	B7	229	D+15	CHL	FLU	TCBS+10µg/mL CHL	19,3
Larvae	B1	232	D+15	CHL	FLU	TCBS	17,2
Larvae	B7	231	D+15	CHL	FLU	TCBS	0,0
Larvae	B1	232	D+15	CHL	FLU	TCBS+10µg/mL CHL	0,0
Larvae	B7	231	D+15	CHL	FLU	TCBS+10µg/mL CHL	0,0
Larvae	B1	218	D+15	CHL	FLO	TCBS	0,0
Larvae	B7	220	D+15	CHL	FLO	TCBS	21,0
Larvae	B1	218	D+15	CHL	FLO	TCBS+10µg/mL CHL	0,0
Larvae	B7	220	D+15	CHL	FLO	TCBS+10µg/mL CHL	0,0
Spat	Pool1	/	D+105	A-	A-	TCBS	2400
Spat	Pool2	/	D+105	A-	A-	TCBS	1750
Spat	Pool3	/	D+105	A-	A-	TCBS	1950
Spat	Pool1	/	D+105	A-	FLO	TCBS	5300
Spat	Pool2	/	D+105	A-	FLO	TCBS	2800
Spat	Pool3	/	D+105	A-	FLO	TCBS	4100
Spat	Pool1	/	D+105	A-	FLU	TCBS	2050
Spat	Pool2	/	D+105	A-	FLU	TCBS	2600
Spat	Pool3	/	D+105	A-	FLU	TCBS	1200
Spat	Pool1	/	D+105	CHL	A-	TCBS	3150
Spat	Pool2	/	D+105	CHL	A-	TCBS	1350
Spat	Pool3	/	D+105	CHL	A-	TCBS	3200
Spat	Pool1	/	D+105	CHL	FLO	TCBS	1900
Spat	Pool2	/	D+105	CHL	FLO	TCBS	750
Spat	Pool3	/	D+105	CHL	FLO	TCBS	3350
Spat	Pool1	/	D+105	CHL	FLU	TCBS	2500
Spat	Pool2	/	D+105	CHL	FLU	TCBS	3250
Spat	Pool3	/	D+105	CHL	FLU	TCBS	6550
Spat	Pool1	/	D+105	A-	A-	TCBS+10µg/mL CHL	0
Spat	Pool2	/	D+105	A-	A-	TCBS+10µg/mL CHL	0
Spat	Pool3	/	D+105	A-	A-	TCBS+10µg/mL CHL	0
Spat	Pool1	/	D+105	A-	FLO	TCBS+10µg/mL CHL	0
Spat	Pool2	/	D+105	A-	FLO	TCBS+10µg/mL CHL	0
Spat	Pool3	/	D+105	A-	FLO	TCBS+10µg/mL CHL	0
Spat	Pool1	/	D+105	A-	FLU	TCBS+10µg/mL CHL	0
Spat	Pool2	/	D+105	A-	FLU	TCBS+10µg/mL CHL	0
Spat	Pool3	/	D+105	A-	FLU	TCBS+10µg/mL CHL	0
Spat	Pool1	/	D+105	CHL	A-	TCBS+10µg/mL CHL	0
Spat	Pool2	/	D+105	CHL	A-	TCBS+10µg/mL CHL	0
Spat	Pool3	/	D+105	CHL	A-	TCBS+10µg/mL CHL	0
Spat	Pool1	/	D+105	CHL	FLO	TCBS+10µg/mL CHL	0
Spat	Pool2	/	D+105	CHL	FLO	TCBS+10µg/mL CHL	0
Spat	Pool3	/	D+105	CHL	FLO	TCBS+10µg/mL CHL	0
Spat	Pool1	/	D+105	CHL	FLU	TCBS+10µg/mL CHL	0
Spat	Pool2	/	D+105	CHL	FLU	TCBS+10µg/mL CHL	0
Spat	Pool3	/	D+105	CHL	FLU	TCBS+10µg/mL CHL	0

Table S6: Pool sequencing analysis

Pool	Sample	Hatchery time	Antibiotic treatment history	Number of isolates	Culture condition	Accession number	Sequencing technology	Total assembly length	Number of contigs	Largest contig	GC %	Confirmed CHL-resistant gene sequences
1	Sperm	D0	CHL	6	TCBS + CHL 10µg/ml 20°C	ERX14309353	NextSeq 550 Illumina	10912109	754	396488	42,56	catA2_P1_N176; catB9_P1_N81_Seq2A; catB9_P1_N8_Seq5
2	Larvae	D+2	A-	11	TCBS + CHL 10µg/ml 20°C	ERX14309351	NextSeq 550 Illumina	18767739	13102	150515	42,65	catA2_P2_N449; catB9_P2_N1001_Seq4; catB9_P2_N255_Seq2B
3	Larvae	D+2	CHL	20	TCBS + CHL 10µg/ml 20°C	ERX14309352	NextSeq 550 Illumina	20505966	16276	134997	42,32	catA2_P3_N430; catB9_P3_N10_Seq2A; catB9_P3_N7_Seq1
4	Larve	D+7	A-/FLU	2	TCBS + CHL 10µg/ml 20°C	ERX14309355	NextSeq 550 Illumina	8710900	8848	413976	43,77	catA2_P4_N59;
5	Larvae	D+7	CHL/FLU et CHL/A-	30 (13+17)	TCBS + CHL 10µg/ml 20°C	ERX14309354	NextSeq 550 Illumina	12972639	4221	265173	43,72	catA2_P5_N265; catB9_P5_N172_Seq6; catB9_P5_N3_Seq1
6	Larvae	D+15	CHL/FLU et CHL/A-	24 (4+20)	TCBS + CHL 10µg/ml 20°C	ERX14309347	NextSeq 550 Illumina	12566021	2760	293681	43,78	catA2_P6_N259; catB9_P6_N10_Seq3; catB9_P6_N2_Seq1
7	Seawater	D-27	A-	1	TCBS + CHL 10µg/ml 20°C	ERX14309344	NextSeq 550 Illumina	5423319	43	466854	44.27	catA2_P7_N125; catA2_P7_N158; catA2_P7_N28; catB9_P7_N85_Seq7
8	Seawater	D-27	CHL	16	TCBS + CHL 10µg/ml 20°C	ERX14309349	NextSeq 550 Illumina	15712321	69588	141305	43,76	catA2_P8_N692; catB9_P8_N569_Seq8; catB9_P8_N150_Seq9
9	Seawater	D-13	CHL	24	TCBS + CHL 10µg/ml 20°C	ERX14309346	NextSeq 550 Illumina	15467347	27593	324167	42,5	catA2_P9_N580; catB9_P9_N502_Seq10; catB9_P9_N9_Seq2A;
10	Seawater	D0	CHL	9	TCBS + CHL 10µg/ml 20°C	ERX14309348	NextSeq 550 Illumina	17688771	29403	110113	42,7	catB9_P10_N1812_Seq11; catB9_P10_N866_Seq12;
11	Seawater	D+2	A-	12	TCBS + CHL 10µg/ml 20°C	ERX14309345	NextSeq 550 Illumina	18000650	33439	227714	42,55	catA2_P11_N270; catB9_P11_N599_Seq13; catB9_P11_N5_Seq2A
12	Seawater	D+2	CHL	20	TCBS + CHL 10µg/ml 20°C	ERX14309350	NextSeq 550 Illumina	26483641	66470	76785	42,62	catA2_P12_N775; catB9_P12_N1726_Seq14; catB9_P12_N167_Seq1

Table S7: ARGs identification and characterisation from pool-sequencing data based on Abricate (resfinder database) and PlasFlow results (CHL: Chloramphenicol).

Confirmed CHL-resistant gene sequences	Pool	Sample	Hatchery time	Antibiotic treatment history	Contig sequence	Start on contig	End on contig	Strand	Gene	Coverage	Gaps	%Coverage	%Identity	Accession	Origin (PlasFLow)	Sequence length	Start codon	Stop codon	Sequence type	Group
catA2_P1_N176	1	Sperm	D0	CHL	NODE_176_length_8569_cov_505.298802	5084	5725	-	*catA2_1*	1-642/642	0/0	100.00	89.56	X53796	Plasmid	642	ATG	TAA	/	G1
catB9_P1_N81_Seq2A	1	Sperm	D0	CHL	NODE_81_length_48148_cov_18.911484	27260	27751	-	*catB9_1*	46-537/630	0/0	78.10	73.17	AF462019	Chromosome	633	TTG	TAA	Seq2A	G2
catB9_P1_N8_Seq5	1	Sperm	D0	CHL	NODE_8_length_201215_cov_94.735743	101400	101955	+	*catB9_1*	44-599/630	0/0	88.25	69.42	AF462019	Chromosome	627	ATG	TAA	Seq5	G4
catA2_P2_N449	2	Larvae	D+2	A-	NODE_449_length_8569_cov_1070.857529	5084	5725	-	*catA2_1*	1-642/642	0/0	100.00	89.56	X53796	Plasmid	642	ATG	TAA	/	G1
catB9_P2_N1001_Seq4	2	Larvae	D+2	A-	NODE_1001_length_4281_cov_230.632040	835	1398	+	*catB9_1*	46-609/630	0/0	89.52	67.55	AF462019	Unidentified	627	ATG	TAA	Seq4	G4
catB9_P2_N255_Seq2B	2	Larvae	D+2	A-	NODE_255_length_12659_cov_35.312996	12091	12582	-	*catB9_1*	46-537/630	0/0	78.10	73.17	AF462019	Chromosome	633	TTG	TAA	Seq2B	G2
catA2_P3_N430	3	Larvae	D+2	CHL	NODE_430_length_8569_cov_983.939276	2845	3486	+	*catA2_1*	1-642/642	0/0	100.00	89.56	X53796	Plasmid	642	ATG	TAA	/	G1
catB9_P3_N10_Seq2A	3	Larvae	D+2	CHL	NODE_10_length_112213_cov_30.945274	109706	110197	-	*catB9_1*	46-537/630	0/0	78.10	73.17	AF462019	Chromosome	633	TTG	TAA	Seq2A	G2
catB9_P3_N7_Seq1	3	Larvae	D+2	CHL	NODE_7_length_118173_cov_87.246254	4728	5151	-	*catB9_1*	44-467/630	0/0	67.30	72.41	AF462019	Chromosome	633	ATG	TAA	Seq1	G3
catA2_P4_N59	4	Larvae	D+7	A-/FLU	NODE_59_length_8569_cov_109.788114	2845	3486	+	*catA2_1*	1-642/642	0/0	100.00	89.56	X53796	Plasmid	642	ATG	TAA	/	G1
catA2_P5_N265	5	Larvae	D+7	CHL/FLU et CHL/A-	NODE_265_length_8569_cov_2060.532300	2845	3486	+	*catA2_1*	1-642/642	0/0	100.00	89.56	X53796	Plasmid	642	ATG	TAA	/	G1
catB9_P5_N172_Seq6	5	Larvae	D+7	CHL/FLU et CHL/A-	NODE_172_length_12069_cov_231.991510	8270	8833	+	*catB9_1*	46-609/630	0/0	89.52	68.97	AF462019	Unidentified	627	ATG	TAA	Seq6	G4
catB9_P5_N3_Seq1	5	Larvae	D+7	CHL/FLU et CHL/A-	NODE_3_length_183994_cov_296.262712	178844	179267	+	*catB9_1*	44-467/630	0/0	67.30	72.41	AF462019	Chromosome	633	ATG	TAA	Seq1	G3
catA2_P6_N259	6	Larvae	D+15	CHL/FLU et CHL/A-	NODE_259_length_8569_cov_1598.501409	5084	5725	-	*catA2_1*	1-642/642	0/0	100.00	89.56	X53796	Plasmid	642	ATG	TAA	/	G1
catB9_P6_N10_Seq3	6	Larvae	D+15	CHL/FLU et CHL/A-	NODE_9_length_155502_cov_103.730384	138156	138577	+	*catB9_1*	46-467/630	0/0	66.98	70.85	AF462019	Chromosome	627	ATG	TAA	Seq3	G4
catB9_P6_N2_Seq1	6	Larvae	D+15	CHL/FLU et CHL/A-	NODE_2_length_274997_cov_388.636531	4728	5151	-	*catB9_1*	44-467/630	0/0	67.30	72.41	AF462019	Chromosome	633	ATG	TAA	Seq1	G3
catA2_P7_N125	7	Seawater	D-27	A-	NODE_125_length_17751_cov_1071.067812	2795	3436	+	*catA2_1*	1-642/642	0/0	100.00	89.56	X53796	Plasmid	642	ATG	TAA	/	G1
catA2_P7_N158	7	Seawater	D-27	A-	NODE_158_length_4456_cov_1468.097705	3278	3919	+	*catA2_1*	1-642/642	0/0	100.00	89.56	X53796	Plasmid	642	ATG	TAA	/	G1

Confirmed CHL-resistant gene sequences	Pool	Sample	Hatchery time	Antibiotic treatment history	Contig sequence	Start on contig	End on contig	Strand	Gene	Coverage	Gaps	%Coverage	%Identity	Accession	Origin (PlasFLow)	Sequence length	Start codon	Stop codon	Sequence type	Group
catB9_P7_N85_Seq7	7	Seawater	D-27	A-	NODE_85_length_39681_cov_473.290870	17313	17734	-	*catB9_1*	46-467/630	0/0	66.98	71.09	AF462019	Unidentified	627	ATG	TAA	Seq7	G4
catA2_P8_N692	8	Seawater	D-27	CHL	NODE_692_length_3897_cov_1170.503904	412	1053	-	*catA2_1*	1-642/642	0/0	100.00	89.56	X53796	Plasmid	642	ATG	TAA	/	G1
catB9_P8_N569_Seq8	8	Seawater	D-27	CHL	NODE_569_length_4398_cov_347.465807	2113	2534	-	*catB9_1*	46-467/630	0/0	66.98	71.56	AF462019	Chromosome	627	ATG	TAA	Seq8	G4
catB9_P8_N150_Seq9	8	Seawater	D-27	CHL	NODE_150_length_10228_cov_222.506832	4682	5173	+	*catB9_1*	46-537/630	0/0	78.10	70.12	AF462019	Unidentified	627	ATG	TAG	Seq9	G4
catA2_P9_N580	9	Seawater	D-13	CHL	NODE_580_length_3973_cov_1249.672282	2795	3436	+	*catA2_1*	1-642/642	0/0	100.00	89.56	X53796	Plasmid	642	ATG	TAA	/	G1
catB9_P9_N502_Seq10	9	Seawater	D-13	CHL	NODE_502_length_4491_cov_375.491885	811	1232	+	*catB9_1*	46-467/630	0/0	66.98	71.09	AF462019	Chromosome	627	ATG	TAA	Seq10	G4
catB9_P9_N9_Seq2A	9	Seawater	D-13	CHL	NODE_9_length_189593_cov_84.753110	20830	21321	+	*catB9_1*	46-537/630	0/0	78.10	73.17	AF462019	Chromosome	633	TTG	TAA	Seq2A	G2
catB9_P10_N1812_Seq11	10	Seawater	D0	CHL	NODE_1812_length_1821_cov_271.301812	1133	1696	+	*catB9_1*	46-609/630	0/0	89.52	67.91	AF462019	Chromosome	627	ATG	TAA	Seq11	G4
catB9_P10_N866_Seq12	10	Seawater	D0	CHL	NODE_866_length_4067_cov_250.692173	405	958	-	*catB9_1*	46-599/630	0/0	87.94	68.23	AF462019	Chromosome	627	ATG	TAA	Seq12	G4
catA2_P11_N270	11	Seawater	D+2	A-	NODE_270_length_8569_cov_1632.819356	2845	3486	+	*catA2_1*	1-642/642	0/0	100.00	89.56	X53796	Plasmid	642	ATG	TAA	/	G1
catB9_P11_N599_Seq13	11	Seawater	D+2	A-	NODE_599_length_4364_cov_480.816663	918	1481	+	*catB9_1*	46-609/630	0/0	89.52	68.08	AF462019	Chromosome	627	ATG	TAA	Seq13	G4
catB9_P11_N5_Seq2A	11	Seawater	D+2	A-	NODE_5_length_153695_cov_28.753332	1872	2363	+	*catB9_1*	46-537/630	0/0	78.10	73.17	AF462019	Chromosome	633	TTG	TAA	Seq2A	G2
catA2_P12_N775	12	Seawater	D+2	CHL	NODE_775_length_5459_cov_1601.057550	2666	3307	-	*catA2_1*	1-642/642	0/0	100.00	89.56	X53796	Plasmid	642	ATG	TAA	/	G1
catB9_P12_N1726_Seq14	12	Seawater	D+2	CHL	NODE_1726_length_2819_cov_262.302098	274	695	-	*catB9_1*	46-467/630	0/0	66.98	71.80	AF462019	Chromosome	627	ATG	TAA	Seq14	G4
catB9_P12_N167_Seq1	12	Seawater	D+2	CHL	NODE_167_length_14817_cov_158.530551	12209	12632	+	*catB9_1*	44-467/630	0/0	67.30	72.41	AF462019	Unidentified	633	ATG	TAA	Seq1	G3

Table S8: Information regarding collected oyster tissues for qPCR quantification

Date of sampling	Site	Days	Sample type	Antibiotic history		Number of individuals	Female/ Male	Sample name
				Broodstock	Larvae			
08/03/2021	Argenton	D0	Adult oyster	CHL	/	1	F	CHL1
08/03/2021	Argenton	D0	Adult oyster	CHL	/	1	F	CHL2
08/03/2021	Argenton	D0	Adult oyster	CHL	/	1	F	CHL3
08/03/2021	Argenton	D0	Adult oyster	CHL	/	1	F	CHL4
08/03/2021	Argenton	D0	Adult oyster	CHL	/	1	M	CHL5
08/03/2021	Argenton	D0	Adult oyster	CHL	/	1	F	CHL6
08/03/2021	Argenton	D0	Adult oyster	CHL	/	1	F	CHL7
08/03/2021	Argenton	D0	Adult oyster	CHL	/	1	M	CHL8
08/03/2021	Argenton	D0	Adult oyster	A-	/	1	F	A-1
08/03/2021	Argenton	D0	Adult oyster	A-	/	1	F	A-2
08/03/2021	Argenton	D0	Adult oyster	A-	/	1	F	A-3
08/03/2021	Argenton	D0	Adult oyster	A-	/	1	F	A-4
08/03/2021	Argenton	D0	Adult oyster	A-	/	1	F	A-5
08/03/2021	Argenton	D0	Adult oyster	A-	/	1	F	A-6
08/03/2021	Argenton	D0	Adult oyster	A-	/	1	F	A-7
08/03/2021	Argenton	D0	Adult oyster	A-	/	1	F	A-8
10/03/2021	Argenton	D+2	D larvae	CHL	/	52930	/	CHL9
10/03/2021	Argenton	D+2	D larvae	CHL	/	52930	/	CHL10
10/03/2021	Argenton	D+2	D larvae	CHL	/	52000	/	CHL11
10/03/2021	Argenton	D+2	D larvae	CHL	/	52000	/	CHL12
10/03/2021	Argenton	D+2	D larvae	CHL	/	54730	/	CHL13
10/03/2021	Argenton	D+2	D larvae	CHL	/	54730	/	CHL14
10/03/2021	Argenton	D+2	D larvae	CHL	/	51464	/	CHL15
10/03/2021	Argenton	D+2	D larvae	CHL	/	51464	/	CHL16
10/03/2021	Argenton	D+2	D larvae	A-	/	50000	/	A-9
10/03/2021	Argenton	D+2	D larvae	A-	/	50000	/	A-10
10/03/2021	Argenton	D+2	D larvae	A-	/	50000	/	A-11
10/03/2021	Argenton	D+2	D larvae	A-	/	50000	/	A-12
10/03/2021	Argenton	D+2	D larvae	A-	/	48880	/	A-13
10/03/2021	Argenton	D+2	D larvae	A-	/	48880	/	A-14
10/03/2021	Argenton	D+2	D larvae	A-	/	52020	/	A-15
10/03/2021	Argenton	D+2	D larvae	A-	/	52020	/	A-16
15/03/2021	Argenton	D+7	D larvae	CHL	A-	50000	/	CHL/A-1
15/03/2021	Argenton	D+7	D larvae	CHL	A-	50000	/	CHL/A-2
15/03/2021	Argenton	D+7	D larvae	CHL	A-	50000	/	CHL/A-3
15/03/2021	Argenton	D+7	D larvae	CHL	A-	50000	/	CHL/A-4
15/03/2021	Argenton	D+7	D larvae	CHL	A-	50000	/	CHL/A-5
15/03/2021	Argenton	D+7	D larvae	CHL	A-	50000	/	CHL/A-6
15/03/2021	Argenton	D+7	D larvae	CHL	A-	50000	/	CHL/A-7
15/03/2021	Argenton	D+7	D larvae	CHL	A-	50000	/	CHL/A-8
15/03/2021	Argenton	D+7	D larvae	A-	A-	50000	/	A-/A-1
15/03/2021	Argenton	D+7	D larvae	A-	A-	50000	/	A-/A-2
15/03/2021	Argenton	D+7	D larvae	A-	A-	50000	/	A-/A-3
15/03/2021	Argenton	D+7	D larvae	A-	A-	50000	/	A-/A-4
15/03/2021	Argenton	D+7	D larvae	A-	A-	50000	/	A-/A-5
15/03/2021	Argenton	D+7	D larvae	A-	A-	50000	/	A-/A-6
15/03/2021	Argenton	D+7	D larvae	A-	A-	50000	/	A-/A-7
15/03/2021	Argenton	D+7	D larvae	A-	A-	50000	/	A-/A-8
23/03/2021	Argenton	D+15	Vegeler larvae	CHL	A-	50000	/	CHL/A-9
23/03/2021	Argenton	D+15	Vegeler larvae	CHL	A-	50000	/	CHL/A-10
23/03/2021	Argenton	D+15	Vegeler larvae	CHL	A-	50000	/	CHL/A-11
23/03/2021	Argenton	D+15	Vegeler larvae	CHL	A-	50000	/	CHL/A-12
23/03/2021	Argenton	D+15	Vegeler larvae	CHL	A-	50000	/	CHL/A-14
23/03/2021	Argenton	D+15	Vegeler larvae	CHL	A-	50000	/	CHL/A-16
23/03/2021	Argenton	D+15	Vegeler larvae	CHL	A-	50000	/	CHL/A-19
23/03/2021	Argenton	D+15	Vegeler larvae	CHL	A-	50000	/	CHL/A-20
23/03/2021	Argenton	D+15	Vegeler larvae	A-	A-	50000	/	A-/A-9
23/03/2021	Argenton	D+15	Vegeler larvae	A-	A-	50000	/	A-/A-10
23/03/2021	Argenton	D+15	Vegeler larvae	A-	A-	50000	/	A-/A-11
23/03/2021	Argenton	D+15	Vegeler larvae	A-	A-	50000	/	A-/A-12
23/03/2021	Argenton	D+15	Vegeler larvae	A-	A-	50000	/	A-/A-13
23/03/2021	Argenton	D+15	Vegeler larvae	A-	A-	50000	/	A-/A-14
23/03/2021	Argenton	D+15	Vegeler larvae	A-	A-	50000	/	A-/A-15
23/03/2021	Argenton	D+15	Vegeler larvae	A-	A-	50000	/	A-/A-16
23/06/2021	Bouin	D+105	Spat	A-	A-	3	/	A-/A- T0-THAU #1
23/06/2021	Bouin	D+105	Spat	A-	A-	3	/	A-/A- T0-THAU #2

Date of sampling	Site	Days	Sample type	Antibiotic history		Number of individuals	Female/ Male	Sample name
				Broodstock	Larvae			
23/06/2021	Bouin	D+105	Spat	A-	A-	3	/	A-/A- T0-THAU #3
23/06/2021	Bouin	D+105	Spat	A-	A-	3	/	A-/A- T0-THAU #4
23/06/2021	Bouin	D+105	Spat	A-	A-	3	/	A-/A- T0-THAU #5
23/06/2021	Bouin	D+105	Spat	A-	A-	3	/	A-/A- T0-THAU #6
23/06/2021	Bouin	D+105	Spat	CHL	A-	3	/	CHL/A- T0-THAU #1
23/06/2021	Bouin	D+105	Spat	CHL	A-	3	/	CHL/A- T0-THAU #2
23/06/2021	Bouin	D+105	Spat	CHL	A-	3	/	CHL/A- T0-THAU #3
23/06/2021	Bouin	D+105	Spat	CHL	A-	3	/	CHL/A- T0-THAU #4
23/06/2021	Bouin	D+105	Spat	CHL	A-	3	/	CHL/A- T0-THAU #5
23/06/2021	Bouin	D+105	Spat	CHL	A-	3	/	CHL/A- T0-THAU #6

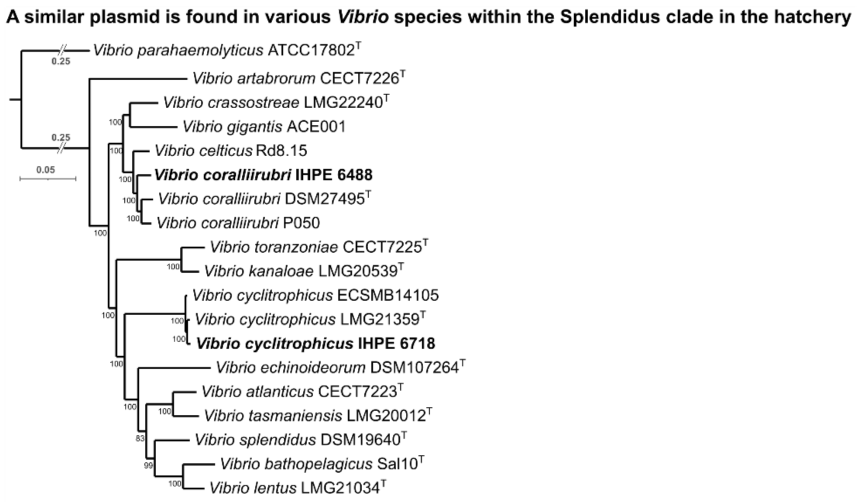

Figure S2: Phylogenetic tree based on core genome analysis (474 genes) of *Vibrio* species in the *Splendidus* clade, rooted using *V. parahaemolyticus*. Species carrying the pAQU-MAN plasmid are in bold. Strain IHPE 6488 clusters with *Vibrio coralliirubri* species, while IHPE 6718 aligns with *Vibrio cyclitrophycus*.

Table S9: Average Nucleotide Identity (ANI) comparisons of bacterial isolates carrying R-plasmids IHPE 6488 and IHPE 6718 with reference *Vibrio* genomes from the NCBI RefSeq database.

**Reference genome**			**ANI value for query genome**	
**Species**	**Strain**	**NCBI RefSeq assembly**	**IHPE 6488**	**IHPE 6718**
V. artabrorum	CECT7226^T	GCF_024347295.1	88.5842	86.6356
V. atlanticus	CECT7223^T	GCF_024347315.1	90.4498	88.7187
V. bathopelagicus	Sal10^T	GCF_014879975.1	88.9043	88.2454
V. celticus	Rd8.15	GCF_002156525.1	**95.3516**	89.2786
V. coralliirubri	P050	GCF_036864355.1	**95.8814**	89.1047
V. coralliirubri	DSM27495^T	GCF_024347375.1	**95.8597**	89.26
V. crassostreae	LMG22240^T	GCF_024347415.1	92.2163	88.772
V. cyclitrophicus	ECSMB14105	GCF_005144905.1	89.1982	**99.0846**
V. cyclitrophicus	LMG21359^T	GCF_024347435.1	89.1208	**98.8763**
V. echinoideorum	DSM107264^T	GCF_024347455.1	88.8153	90.0425
V. gigantis	ACE001	GCF_022371215.1	90.6631	88.1361
V. kanaloae	LMG20539^T	GCF_024347535.1	89.5917	88.0119
V. lentus	LMG21034^T	GCF_024347555.1	89.4974	88.7624
V. parahaemolyticus	ATCC17802^T	GCF_001558495.2	80.161	80.272
V. splendidus	DSM19640^T	GCF_002737025.1	91.6501	89.4426
V. tasmaniensis	LMG20012^T	GCF_024347635.1	89.9338	88.6608
V. toranzoniae	CECT7225^T	GCF_024347655.1	88.3896	87.8613

Table S10: Gene annotation of the pAQU-MAN_1 plasmid according to Bakta (Prodigal).

Locus tag	Gene	Function	Start	End	Strand
VCR34_0001	*pseudogene*	Replication protein	1	1086	+
VCR34_0002		DUF6531 domain-containing protein	1584	5822	+
VCR34_0003		hypothetical protein	5797	6258	+
VCR34_0004		hypothetical protein	6326	6757	+
VCR34_0005		RHS repeat-associated core domain-containing protein	6809	7693	+
VCR34_0006		hypothetical protein	7722	7979	+
VCR34_0007		DUF3955 domain-containing protein	8149	8403	+
VCR34_0008		RHS repeat-associated core domain-containing protein	8403	9197	+
VCR34_0009		hypothetical protein	9316	9543	+
VCR34_0010		hypothetical protein	9620	10144	+
VCR34_0011		hypothetical protein	10349	10777	+
VCR34_0012		Lipoprotein	10861	11307	+
VCR34_0013		hypothetical protein	11577	11786	+
VCR34_0014		ATPase domain-containing protein	11823	12917	-
VCR34_0015		hypothetical protein	13149	13391	+
VCR34_0016		hypothetical protein	13411	13671	+
VCR34_0017		NERD domain-containing protein	13728	14705	+
VCR34_0018		hypothetical protein	14799	15554	+
VCR34_0019		HTH OST-type domain-containing protein	15572	16339	+
VCR34_0020		methyl-accepting chemotaxis protein	16648	18201	-
VCR34_0021	*parA*	ParA family protein	18963	19769	+
VCR34_0022		ParB/Sulfiredoxin domain-containing protein	19771	20961	+
VCR34_0023		Helix-turn-helix domain-containing protein	20974	21246	+
VCR34_0024		RNA-binding protein	21387	21662	+
VCR34_0025		hypothetical protein	21680	21982	+
VCR34_0026		hypothetical protein	22076	22165	+
VCR34_0027	*pseudogene*	hypothetical protein	22158	22268	+
VCR34_0028		Transcriptional regulator	22287	22718	+
VCR34_0029		GtrA-like protein domain-containing protein	22715	22960	+
VCR34_0030		Holin	22957	23313	+
VCR34_0031		hypothetical protein	23303	24055	+
VCR34_0032		MFS transporter	24074	24367	+
VCR34_0033		hypothetical protein	24381	24584	+
VCR34_0034		hypothetical protein	24581	24802	+
VCR34_0035		VRR-NUC domain-containing protein	24889	25179	+
VCR34_0036	*tnp*	ISAs1 family ISVha1 transposase	25249	26370	+
VCR34_0037		Ribosome modulation factor	26583	26816	+
VCR34_0038		Integrase	26878	28839	+
VCR34_0039		DUF262 domain-containing protein	28836	29429	+
VCR34_0040		hypothetical protein	29439	30053	+
VCR34_0041	*tnp*	IS5 family ISVba2 transposase	30818	31540	+
VCR34_0042		hypothetical protein	31774	32496	-
VCR34_0043	*topB*	DNA topoisomerase	32609	34816	-
VCR34_0044		hypothetical protein	34817	35119	-
VCR34_0045		Lipoprotein	35101	35889	-
VCR34_0046		hypothetical protein	35882	36121	-
VCR34_0047	*mobH*	MobH family relaxase	36285	39359	+
VCR34_0048	*traD*	Type IV conjugative transfer system protein TraD	39366	41234	+
VCR34_0049		Conjugative transfer protein 234	41244	41828	+
VCR34_0050		DUF4400 domain-containing protein	41785	42414	+
VCR34_0051		Lipoprotein	42431	42832	+
VCR34_0052		hypothetical protein	43001	43144	+
VCR34_0053	*traL*	type IV conjugative transfer system protein TraL	43302	43583	+
VCR34_0054		Pilus assembly protein	43580	44209	+
VCR34_0055	*traK*	Conjugal transfer protein TraK	44199	45149	+
VCR34_0056	*traB*	Conjugal transfer protein TraB	45153	46502	+
VCR34_0057	*traV*	type IV conjugative transfer system lipoprotein TraV	46559	47050	+
VCR34_0058	*traA*	Conjugal transfer protein TraA	47065	47439	+
VCR34_0059	*traA*	Conjugal transfer protein TraA	47534	47908	+
VCR34_0060		DUF4165 domain-containing protein	48116	53536	+
VCR34_0061		Thiol:disulfide interchange protein	53692	54396	+
VCR34_0062	*traC*	type IV secretion system protein TraC	54393	56843	+
VCR34_0063		Conjugative transfer protein 345	56861	57181	+
VCR34_0064		Signal peptidase I	57178	57693	+
VCR34_0065	*traW*	Conjugal transfer protein TraW	57656	58924	+
VCR34_0066		EAL domain-containing protein	58921	59625	+
VCR34_0067	*traU*	Conjugal transfer protein TraU	59622	60629	+
VCR34_0068	*traN*	conjugal transfer mating pair stabilization protein TraN	60825	63605	+

Locus tag	Gene	Function	Start	End	Strand
VCR34_0069		hypothetical protein	63833	65161	+
VCR34_0070		DUF1845 domain-containing protein	65262	65936	-
VCR34_0071		hypothetical protein	66233	66556	+
VCR34_0072		hypothetical protein	66722	66943	+
VCR34_0073		hypothetical protein	67005	67136	+
VCR34_0074		hypothetical protein	67206	67523	+
VCR34_0075		Phage protein	67896	68072	+
VCR34_0076		CbbQ/NirQ/NorQ/GpvN family protein	68286	69257	+
VCR34_0077		hypothetical protein	69267	70148	+
VCR34_0078	*bet*	phage recombination protein Bet	70214	71218	+
VCR34_0079		Endonuclease	71279	72289	+
VCR34_0080		hypothetical protein	72375	72518	+
VCR34_0081		hypothetical protein	72738	72881	+
VCR34_0082		DUF3150 domain-containing protein	72965	74269	+
VCR34_0083		VWA domain-containing protein	74338	76098	+
VCR34_0084		hypothetical protein	76207	76701	+
VCR34_0085		hypothetical protein	76982	78142	+
VCR34_0086		hypothetical protein	78214	78396	+
VCR34_0087		DUF3085 domain-containing protein	78650	79129	+
VCR34_0088	*yubB*	YubB ferredoxin-like domain-containing protein	79188	79685	+
VCR34_0089		DNA adenine modification methylase	79729	80619	+
VCR34_0090	*yfaE*	class I ribonucleotide reductase maintenance protein YfaE	80926	81150	+
VCR34_0091		Phage protein	81212	81829	+
VCR34_0092		hypothetical protein	81816	82100	+
VCR34_0093		hypothetical protein	82132	82323	+
VCR34_0094		Topoisomerase II	82407	82817	+
VCR34_0095		hypothetical protein	82873	83508	+
VCR34_0096		hypothetical protein	83722	84108	+
VCR34_0097	*tusA*	sulfurtransferase TusA	84139	84369	+
VCR34_0098		hypothetical protein	84431	84745	+
VCR34_0099		hypothetical protein	84835	85014	+
VCR34_0100		hypothetical protein	85062	85307	+
VCR34_0101		hypothetical protein	85368	86285	+
VCR34_0102		hypothetical protein	86512	86805	+
VCR34_0103		hypothetical protein	86882	87082	+
VCR34_0104		Phage protein	87094	87507	+
VCR34_0105		hypothetical protein	87586	88047	+
VCR34_0106		Transmembrane protein	88237	88668	+
VCR34_0107		hypothetical protein	88736	88984	+
VCR34_0108		hypothetical protein	89061	89177	+
VCR34_0109	*yfbR*	5'-deoxynucleotidase	89660	90499	+
VCR34_0110		4-diphosphocytidyl-2C-methyl-D-erythritol kinase	90496	90729	+
VCR34_0111		hypothetical protein	90813	91199	+
VCR34_0112	*ngrC*	NgrC	91286	92191	+
VCR34_0113		hypothetical protein	92274	92831	+
VCR34_0114		hypothetical protein	93051	93611	+
VCR34_0115		Bacteriophage T7 Gp4 DNA primase/helicase N-terminal domain-containing protein	93761	95539	+
VCR34_0116		hypothetical protein	95526	95771	+
VCR34_0117		hypothetical protein	95852	96403	+
VCR34_0118		hypothetical protein	96481	96921	+
VCR34_0119		Large polyvalent protein-associated domain-containing protein	97294	98748	+
VCR34_0120		Transcriptional regulator	98751	99044	-
VCR34_0121		hypothetical protein	100033	100239	+
VCR34_0122		HNH endonuclease	100243	100524	+
VCR34_0123		Tyrosine specific protein phosphatases domain-containing protein	100675	101223	+
VCR34_0124		Integrase	101281	102249	+
VCR34_0125		Integral membrane protein	102251	103039	+
VCR34_0126	*csgE*	Curli production assembly/transport component CsgE	103036	103524	-
VCR34_0127		hypothetical protein	103527	104228	-
VCR34_0128		Tn3 family transposase	104370	107348	+
VCR34_0129	*tnp*	IS5 family ISKpn13 transposase	107728	108660	-
VCR34_0130	*catA2*	type A-2 chloramphenicol O-acetyltransferase CatII	109334	109975	-
VCR34_0131		Transposase	110035	110688	-
VCR34_0132		Transposase Tn3 family protein	111005	111346	+
VCR34_0133		hypothetical protein	111506	112243	+
VCR34_0134	*Dbxref=RFAM:RF00240,SO:0000655*	RNA-OUT	112241	112310	-
VCR34_0135	*tnp*	IS4 family IS10R transposase	112303	113511	+
VCR34_0136	*lysR*	DNA-binding transcriptional regulator%2C LysR family	113521	113646	-
VCR34_0137	*ydhA*	YdhA protein	113877	114092	-

Locus tag	Gene	Function	Start	End	Strand
VCR34_0138	*tnp*	IS4 family IS10R transposase	114067	115275	-
VCR34_0139	*Dbxref=RFAM:RF 00240,SO:0000655*	RNA-OUT	115268	115337	+
VCR34_0140		hypothetical protein	115335	116072	-
VCR34_0141		Transposase Tn3 family protein	116232	116573	-
VCR34_0142		Transposase	116890	117543	+
VCR34_0143	*catA2*	type A-2 chloramphenicol O-acetyltransferase CatII	117603	118244	+
VCR34_0144	*tnp*	IS5 family ISKpn13 transposase	118918	119850	+
VCR34_0145		Tn3 family transposase	120230	123208	-
VCR34_0146		Sodium/glutamate symporter	123312	123635	-
VCR34_0147	*hmoA*	Heme-degrading monooxygenase HmoA and related ABM domain proteins	124079	124399	+
VCR34_0148		ACT domain-containing protein	124392	124778	+
VCR34_0149	*arsR*	DNA-binding transcriptional regulator%2C ArsR family	124786	125472	+
VCR34_0150	*tetR(B)*	tetracycline resistance transcriptional repressor TetR(B)	125450	126073	-
VCR34_0151	*tet(B)*	tetracycline efflux MFS transporter Tet(B)	126155	127360	+
VCR34_0152		Tn3 family transposase	127723	130743	+
VCR34_0153	*tetC*	tetracyline resistance-associated transcriptional repressor TetC	130751	131374	-
VCR34_0154	*tetD*	Transposon Tn10 TetD protein	131387	131803	+
VCR34_0155	*tnp*	IS4 family IS10R transposase	131813	133021	-
VCR34_0156	*Dbxref=RFAM:RF 00240,SO:0000655*	RNA-OUT	133014	133083	+
VCR34_0157		Restriction endonuclease subunit M	133132	133779	-
VCR34_0158		DNA replication protein	134121	135014	+
VCR34_0159		hypothetical protein	135082	136164	-
VCR34_0160		hypothetical protein	136550	137365	-
VCR34_0161		Calx-beta domain-containing protein	137716	139353	+
VCR34_0162		DUF1566 domain-containing protein	139353	139904	+
VCR34_0163		DNA 3'-5' helicase	139961	141475	+
VCR34_0164	*traF*	Conjugal transfer protein TraF	141565	142599	+
VCR34_0165	*traH*	Conjugal transfer protein TraH	142601	144040	+
VCR34_0166	*traG*	Conjugal transfer protein TraG	144047	147688	+
VCR34_0167		Permease	147739	148125	-
VCR34_0168		Transglycosylase	149131	149673	+
VCR34_0169		Transcriptional regulator	149675	150280	+
VCR34_0170		Regulator	150270	150845	+
VCR34_0171		hypothetical protein	150922	151197	-
VCR34_0172		ParM/StbA family protein	151211	152188	-
VCR34_0173	*mobI*	MobI	152661	153215	+
VCR34_0174		hypothetical protein	153240	153530	+
VCR34_0175		Putative membrane protein	154394	154855	-
VCR34_0176		Lipoprotein	155217	156068	+
VCR34_0177		hypothetical protein	156123	156512	+
VCR34_0178	*mcjB*	Microcin J25-processing protein McjB C-terminal domain-containing protein	156502	156906	+
VCR34_0179		PD-(D/E)XK endonuclease-like domain-containing protein	157080	157601	+
VCR34_0180		hypothetical protein	157683	158156	+
VCR34_0181		hypothetical protein	158167	158460	+
VCR34_0182		hypothetical protein	158466	159470	+
VCR34_0183	*sppA*	Signal peptide peptidase SppA	159586	160548	+
VCR34_0184		Protein-disulfide isomerase	160561	161436	+
VCR34_0185		Lipoprotein	161455	163029	+
VCR34_0186		hypothetical protein	163214	163636	+
VCR34_0187		TNase-like domain-containing protein	163647	164156	+
VCR34_0188		hypothetical protein	164153	164668	+
VCR34_0189		hypothetical protein	164673	164948	-
VCR34_0190		Methyltransferase	165328	166581	+
VCR34_0191		hypothetical protein	166607	166849	+
VCR34_0192		hypothetical protein	166855	167061	+
VCR34_0193		Phage protein	167061	167504	+
VCR34_0194		hypothetical protein	167506	167688	+
VCR34_0195		Permease	167695	168069	-
VCR34_0196	*pseudogene*	Replication protein	168419	169522	+

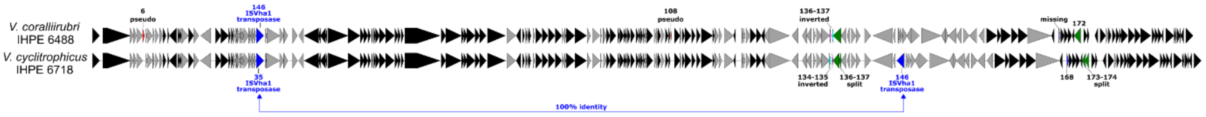

Figure S3: The pAQU-MAN plasmids identified within strain IHPE 6488 and IHPE 6718 were 99.99% similar; only a few differences highlighted in this figure were observed. Two unannotated pseudogenes on IHPE 6718 (IHPE 6488-6 and IHPE 6488-108). One inversion at the junction of the repeat (IHPE 6488-136 and IHPE 6488-137); this is the complex region to be sequenced. One gene not detected by Bakkta (IHPE 6718 -168) but showing 100% nucleic similarity in IHPE 6488. Two splits potentially caused by a misread base by Nanopore (IHPE 6718-136/ IHPE 6718-137 and IHPE 6718-173/ IHPE 6718-174). One additional transposase in IHPE 6718 (IHPE 6718-146) that is 100% identical to its paralog (IHPE 6718-35).

Table S11: Identification and characterisation of ARGs from published R-plasmids used for comparison, using Abricate tool (resfinder database) with identity and coverage set at 80 and 70% respectively.

Strain	Plasmid	Plasmid ID	Start	End	Strand	Locus tag	Gene	Coverage	Gaps	%Coverage	%Identity	Accession
Photobacterium damselae subsp. damselae 04Ya311	pAQU1	AP026782.1	139141	139956	+	PDA35_0158	sul2_2	1-816/816	0/0	100	100	AY034138
Photobacterium damselae subsp. damselae 04Ya311	pAQU1	AP026782.1	140244	141467	+	PDA35_0159	mef(C)_1	1-1224/1224	0/0	100	100	AB571865
Photobacterium damselae subsp. damselae 04Ya311	pAQU1	AP026782.1	141472	142356	+	PDA35_0160	mph(G)_1	1-885/885	0/0	100	100	AB571865
Photobacterium damselae subsp. damselae 04Ya311	pAQU1	AP026782.1	144568	145781	-	PDA35_0163	floR_2	1-1214/1215	0/0	99,92	98,11	AF118107
Photobacterium damselae subsp. damselae 04Ya311	pAQU1	AP026782.1	154748	156667	-	PDA35_0172	tet(M)_8	1-1920/1920	0/0	100	99,74	X04388
Photobacterium damselae subsp. damselae 04Ya311	pAQU1	AP026782.1	159322	160527	-	PDA35_0178	tet(B)_1	1-1206/1206	0/0	100	99,83	AP000342
Vibrio alfacsensis VA-1	plas2	CP140105.1	86700	87356	-	VAL37_0083	qnrS2_1	1-657/657	0/0	100	100	DQ485530
Vibrio alfacsensis VA-1	plas2	CP140105.1	144886	145701	+	VAL37_0158	sul2_2	1-816/816	0/0	100	100	AY034138
Vibrio alfacsensis VA-1	plas2	CP140105.1	156132	156998	-	VAL37_0172	sul1_5	1-867/867	0/0	100	99,89	EU780013
Vibrio alfacsensis VA-1	plas2	CP140105.1	165244	167163	-	VAL37_0182	tet(M)_8	1-1920/1920	0/0	100	99,74	X04388
Vibrio alfacsensis VA-1	plas2	CP140105.1	169612	170817	-	VAL37_0188	tet(B)_1	1-1206/1206	0/0	100	99,92	AP000342
Vibrio alginolyticus	pVAS19	KX957968.1	110378	111244	-	VAL08_0128	sul1_5	1-867/867	0/0	100	99,89	EU780013
Vibrio alginolyticus	pVAS19	KX957968.1	116163	117089	-	VAL08_0135	blaPER-1_1	1-927/927	0/0	100	100	GU944725
Vibrio alginolyticus	pVAS19	KX957968.1	119364	120230	-	VAL08_0137	sul1_5	1-867/867	0/0	100	99,89	EU780013
Vibrio alginolyticus	pVAS19	KX957968.1	120708	121509	-	VAL08_0139	aadA2_1	18-819/819	0/0	97,92	98,63	NC_010870
Vibrio alginolyticus	pVAS19	KX957968.1	121953	122426	-	VAL08_0141	dfrA1_8	1-474/474	0/0	100	99,58	X00926
Vibrio alginolyticus	pVAS114	KX957969.1	123023	123838	+	VAL18_0144	sul2_2	1-816/816	0/0	100	100	AY034138
Vibrio alginolyticus	pVAS19	KX957968.1	123956	124510	-	VAL08_0144	aac(6')-IIa_1	1-555/555	0/0	100	99,64	M29695
Vibrio alginolyticus	pVAS114	KX957969.1	126758	127960	+	VAL18_0148	tet(59)_1	1-1203/1203	0/0	100	99,67	KU736878
Vibrio alginolyticus	pVAS114	KX957969.1	134176	135036	-	VAL18_0152	blaTEM-1B_1	1-861/861	0/0	100	100	AY458016
Vibrio alginolyticus	pVAS19	KX957968.1	134679	135884	-	VAL08_0158	tet(B)_1	1-1206/1206	0/0	100	100	AP000342
Vibrio alginolyticus	pVAS114	KX957969.1	140008	140868	-	VAL18_0157	aac(3)-IId_1	1-861/861	0/0	100	99,88	EU022314
Vibrio alginolyticus	pVAS19	KX957968.1	142650	143306	+	VAL08_0167	qnrVC6_1	1-657/657	0/0	100	100	KC202804
Vibrio alginolyticus	pVAS114	KX957969.1	143175	143717	+	VAL18_0160	ARR-3_4	1-543/543	0/0	100	99,45	FM207631
Vibrio alginolyticus	pVAS114	KX957969.1	143850	144323	+	VAL18_0161	dfrA27_1	1-474/474	0/0	100	100	FJ459817
Vibrio alginolyticus	pVAS114	KX957969.1	144816	145682	+	VAL18_0163	sul1_5	1-867/867	0/0	100	99,89	EU780013
Vibrio alginolyticus	pVAS114	KX957969.1	147968	148894	+	VAL18_0165	blaPER-1_1	1-927/927	0/0	100	100	GU944725
Vibrio alginolyticus	pVAS114	KX957969.1	153813	154679	+	VAL18_0172	sul1_5	1-867/867	0/0	100	99,89	EU780013
Vibrio alginolyticus	pVAS114	KX957969.1	161301	162222	-	VAL18_0180	mph(A)_2	1-921/921	1/1	100	99,67	U36578
Vibrio alginolyticus	pVAS114	KX957969.1	164834	165718	-	VAL18_0184	mph(E)_1	1-885/885	0/0	100	100	DQ839391
Vibrio alginolyticus	pVAS114	KX957969.1	165774	167249	-	VAL18_0185	msr(E)_1	1-1476/1476	0/0	100	100	FR751518
Vibrio alginolyticus 704	pC704	OP958859.1	22606	23262	+	VAL07_0024	qnrS2_1	1-657/657	0/0	100	100	DQ485530
Vibrio alginolyticus 704	pC704	OP958859.1	112404	113219	+	VAL07_0135	sul2_2	1-816/816	0/0	100	100	AY034138
Vibrio alginolyticus 704	pC704	OP958859.1	121333	122199	-	VAL07_0146	sul1_5	1-867/867	0/0	100	99,89	EU780013
Vibrio alginolyticus 704	pC704	OP958859.1	123119	123760	-	VAL07_0147	catA2_1	1-642/642	0/0	100	99,69	X53796
Vibrio alginolyticus 704	pC704	OP958859.1	126457	127323	-	VAL07_0151	sul1_5	1-867/867	0/0	100	99,89	EU780013
Vibrio alginolyticus 704	pC704	OP958859.1	127801	128602	-	VAL07_0153	ant(3'')-Ia_1	171-972/972	0/0	82,51	99,75	X02340

Strain	Plasmid	Plasmid ID	Start	End	Strand	Locus tag	Gene	Coverage	Gaps	%Coverage	%Identity	Accession
Vibrio alginolyticus 704	pC704	OP958859.1	128710	129532	-	VAL07_0154	blaCARB-12_1	45-867/867	0/0	94,93	100	D13210
Vibrio alginolyticus 704	pC704	OP958859.1	129722	130462	+	VAL07_0155	blaVMB-2_1	1-741/741	0/0	100	100	MN865127
Vibrio alginolyticus 704	pC704	OP958859.1	133012	133878	-	VAL07_0158	sul1_5	1-867/867	0/0	100	99,89	EU780013
Vibrio alginolyticus 704	pC704	OP958859.1	142289	143494	+	VAL07_0169	tet(B)_1	1-1206/1206	0/0	100	99,92	AP000342
Vibrio alginolyticus C1579	pC1579	MN865127.1	18315	18849	+	VAL45_0017	qnrVC6_1	123-657/657	0/0	81,43	100	KC202804
Vibrio alginolyticus C1579	pC1579	MN865127.1	19475	19948	+	VAL45_0018	dfrA6_2	1-474/474	0/0	100	99,37	FJ905898
Vibrio alginolyticus C1579	pC1579	MN865127.1	122119	123332	+	VAL45_0128	floR_2	1-1214/1215	0/0	99,92	98,19	AF118107
Vibrio alginolyticus C1579	pC1579	MN865127.1	124289	125125	-	VAL45_0131	aph(6)-Id_1	1-837/837	0/0	100	100	M28829
Vibrio alginolyticus C1579	pC1579	MN865127.1	125125	125927	-	VAL45_0132	aph(3")-Ib_2	2-804/804	0/0	99,88	100	AF024602
Vibrio alginolyticus C1579	pC1579	MN865127.1	125989	126804	-	VAL45_0133	sul2_15	1-816/816	0/0	100	100	FJ968160
Vibrio alginolyticus C1579	pC1579	MN865127.1	158661	159878	+	VAL45_0167	tet(E)_3	1-1218/1218	0/0	100	99,26	CP000645
Vibrio alginolyticus C1579	pC1579	MN865127.1	162930	163796	-	VAL45_0171	sul1_5	1-867/867	0/0	100	99,89	EU780013
Vibrio alginolyticus C1579	pC1579	MN865127.1	164227	165072	-	VAL45_0173	aadA16_1	1-846/846	0/0	100	99,64	EU675686
Vibrio alginolyticus C1579	pC1579	MN865127.1	165253	165726	-	VAL45_0174	dfrA27_1	1-474/474	0/0	100	100	FJ459817
Vibrio alginolyticus C1579	pC1579	MN865127.1	165859	166401	-	VAL45_0175	ARR-3_4	1-543/543	0/0	100	99,45	FM207631
Vibrio alginolyticus C1579	pC1579	MN865127.1	172339	173079	-	VAL45_0180	blaVMB-2_1	1-741/741	0/0	100	100	MN865127
Vibrio alginolyticus C1579	pC1579	MN865127.1	173269	174091	+	VAL45_0181	blaCARB-12_1	45-867/867	0/0	94,93	100	D13210
Vibrio alginolyticus C1579	pC1579	MN865127.1	174199	174993	+	VAL45_0182	ant(3")-Ia_1	171-965/972	0/0	81,79	99,75	X02340
Vibrio alginolyticus C1579	pC1579	MN865127.1	180350	181090	-	VAL45_0186	blaVMB-2_1	1-741/741	0/0	100	100	MN865127
Vibrio alginolyticus C1579	pC1579	MN865127.1	181280	182102	+	VAL45_0187	blaCARB-12_1	45-867/867	0/0	94,93	100	D13210
Vibrio alginolyticus C1579	pC1579	MN865127.1	182210	183004	+	VAL45_0188	ant(3")-Ia_1	171-965/972	0/0	81,79	99,75	X02340
Vibrio alginolyticus C1579	pC1579	MN865127.1	188361	189101	-	VAL45_0192	blaVMB-2_1	1-741/741	0/0	100	100	MN865127
Vibrio alginolyticus C1579	pC1579	MN865127.1	189291	190113	+	VAL45_0193	blaCARB-12_1	45-867/867	0/0	94,93	100	D13210
Vibrio alginolyticus C1579	pC1579	MN865127.1	190221	191015	+	VAL45_0194	ant(3")-Ia_1	171-965/972	0/0	81,79	99,75	X02340
Vibrio alginolyticus C1579	pC1579	MN865127.1	196372	197112	-	VAL45_0198	blaVMB-2_1	1-741/741	0/0	100	100	MN865127
Vibrio alginolyticus C1579	pC1579	MN865127.1	197302	198124	+	VAL45_0199	blaCARB-12_1	45-867/867	0/0	94,93	100	D13210
Vibrio alginolyticus C1579	pC1579	MN865127.1	198232	199026	+	VAL45_0200	ant(3")-Ia_1	171-965/972	0/0	81,79	99,75	X02340
Vibrio coralliirubri IHPE 6488	pAQU-MAN_1	ERX14309694	109334	109975	-	VCR34_0130	catA2_1	1-642/642	0/0	100	89,56	X53796
Vibrio coralliirubri IHPE 6488	pAQU-MAN_1	ERX14309694	117603	118244	+	VCR34_0143	catA2_1	1-642/642	0/0	100	89,56	X53796
Vibrio coralliirubri IHPE 6488	pAQU-MAN_1	ERX14309694	126155	127360	+	VCR34_0151	tet(B)_1	1-1206/1206	0/0	100	99,83	AP000342
Vibrio furnissii MT14	pMT14	CP115190.1	22703	23359	+	VFU19_0024	qnrVC6_1	1-657/657	0/0	100	100	KC202804
Vibrio furnissii MT14	pMT14	CP115190.1	23985	24458	+	VFU19_0025	dfrA31_1	1-474/474	0/0	100	99,58	AB200915
Vibrio furnissii MT14	pMT14	CP115190.1	88328	88984	-	VFU19_0091	qnrS2_1	1-657/657	0/0	100	100	DQ485530
Vibrio furnissii MT14	pMT14	CP115190.1	151101	151916	+	VFU19_0168	sul2_2	1-816/816	0/0	100	100	AY034138
Vibrio furnissii MT14	pMT14	CP115190.1	154836	156038	+	VFU19_0172	tet(59)_1	1-1203/1203	0/0	100	99,58	KU736878
Vibrio harveyi 345	p345-185	CP025539.1	22944	23600	+	VHA15_0023	qnrVC6_1	1-657/657	0/0	100	100	KC202804
Vibrio harveyi 345	p345-185	CP025539.1	24226	24699	+	VHA15_0024	dfrA6_2	1-474/474	0/0	100	99,37	FJ905898
Vibrio harveyi 345	p345-185	CP025539.1	120731	121546	+	VHA15_0143	sul2_2	1-816/816	0/0	100	100	AY034138
Vibrio harveyi 345	p345-185	CP025539.1	124051	125263	-	VHA15_0146	floR_2	1-1214/1215	1/1	99,84	98,11	AF118107
Vibrio harveyi 345	p345-185	CP025539.1	128944	130863	-	VHA15_0153	tet(M)_8	1-1920/1920	0/0	100	99,74	X04388

Strain	Plasmid	Plasmid ID	Start	End	Strand	Locus tag	Gene	Coverage	Gaps	%Coverage	%Identity	Accession
Vibrio harveyi 345	p345-185	CP025539.1	133321	134526	-	VHA15_0159	tet(B)_1	1-1206/1206	0/0	100	99,92	AP000342
Vibrio owensii 20160513VC2W	p2	CP030801.1	21684	22340	+	VOW01_0025	qnrVC6_1	1-657/657	0/0	100	100	KC202804
Vibrio owensii 20160513VC2W	p2	CP030801.1	22966	23439	+	VOW01_0026	dfrA6_2	1-474/474	0/0	100	99,37	FJ905898
Vibrio owensii 20160513VC2W	p2	CP030801.1	119283	120098	+	VOW01_0144	sul2_2	1-816/816	0/0	100	100	AY034138
Vibrio owensii 20160513VC2W	p2	CP030801.1	120386	121257	+	VOW01_0145	mef(C)_1	1-872/1224	0/0	71,24	100	AB571865
Vibrio owensii 20160513VC2W	p2	CP030801.1	124419	126338	-	VOW01_0149	tet(M)_8	1-1920/1920	0/0	100	99,74	X04388
Vibrio owensii 20160513VC2W	p2	CP030801.1	129669	130874	+	VOW01_0157	tet(B)_1	1-1206/1206	0/0	100	99,92	AP000342
Vibrio parahaemolyticus	pVPS62	KX957971.1	22944	23600	+	VPA39_0023	qnrVC6_1	1-657/657	0/0	100	100	KC202804
Vibrio parahaemolyticus	pVPS62	KX957971.1	24226	24699	+	VPA39_0024	dfrA6_2	1-474/474	0/0	100	99,37	FJ905898
Vibrio parahaemolyticus	pVPS43	KX957970.1	115103	115918	+	VPA42_0134	sul2_2	1-816/816	0/0	100	100	AY034138
Vibrio parahaemolyticus	pVPS62	KX957971.1	120731	121546	+	VPA39_0143	sul2_2	1-816/816	0/0	100	100	AY034138
Vibrio parahaemolyticus	pVPS43	KX957970.1	122373	123233	-	VPA42_0140	blaTEM-1B_1	1-861/861	0/0	100	100	AY458016
Vibrio parahaemolyticus	pVPS62	KX957971.1	124050	125263	-	VPA39_0147	floR_2	1-1214/1215	0/0	99,92	98,11	AF118107
Vibrio parahaemolyticus	pVPS43	KX957970.1	128205	129065	-	VPA42_0145	aac(3)-IId_1	1-861/861	0/0	100	99,88	EU022314
Vibrio parahaemolyticus	pVPS62	KX957971.1	128334	130253	-	VPA39_0150	tet(M)_8	1-1920/1920	0/0	100	99,74	X04388
Vibrio parahaemolyticus	pVPS43	KX957970.1	131373	131915	+	VPA42_0148	ARR-3_4	1-543/543	0/0	100	99,45	FM207631
Vibrio parahaemolyticus	pVPS43	KX957970.1	132048	132521	+	VPA42_0149	dfrA27_1	1-474/474	0/0	100	100	FJ459817
Vibrio parahaemolyticus	pVPS62	KX957971.1	132711	133916	-	VPA39_0156	tet(B)_1	1-1206/1206	0/0	100	99,92	AP000342
Vibrio parahaemolyticus	pVPS43	KX957970.1	133014	133880	+	VPA42_0151	sul1_5	1-867/867	0/0	100	99,89	EU780013
Vibrio parahaemolyticus	pVPS43	KX957970.1	136166	137092	+	VPA42_0153	blaPER-1_1	1-927/927	0/0	100	100	GU944725
Vibrio parahaemolyticus	pVPS43	KX957970.1	142011	142877	+	VPA42_0160	sul1_5	1-867/867	0/0	100	99,89	EU780013
Vibrio parahaemolyticus	pVPS43	KX957970.1	149499	150420	-	VPA42_0167	mph(A)_2	1-921/921	1/1	100	99,67	U36578
Vibrio parahaemolyticus	pVPS43	KX957970.1	153032	153916	-	VPA42_0171	mph(E)_1	1-885/885	0/0	100	100	DQ839391
Vibrio parahaemolyticus	pVPS43	KX957970.1	153972	155447	-	VPA42_0172	msr(E)_1	1-1476/1476	0/0	100	100	FR751518
Vibrio parahaemolyticus 2011VPH2	pVPH2	KP791968.1	131854	132720	-	VPA06_0166	sul1_5	1-867/867	0/0	100	99,89	EU780013
Vibrio parahaemolyticus 2011VPH2	pVPH2	KP791968.1	137639	138565	-	VPA06_0173	blaPER-1_1	1-927/927	0/0	100	100	GU944725
Vibrio parahaemolyticus 2011VPH2	pVPH2	KP791968.1	140837	141703	-	VPA06_0176	sul1_5	1-867/867	0/0	100	99,89	EU780013
Vibrio parahaemolyticus 2011VPH2	pVPH2	KP791968.1	142181	142982	-	VPA06_0178	aadA2_1	18-819/819	0/0	97,92	98,63	NC_010870
Vibrio parahaemolyticus 2011VPH2	pVPH2	KP791968.1	143426	143899	-	VPA06_0180	dfrA1_8	1-474/474	0/0	100	99,58	X00926
Vibrio parahaemolyticus 2011VPH2	pVPH2	KP791968.1	145429	145983	-	VPA06_0183	aac(6')-IIa_1	1-555/555	0/0	100	99,64	M29695
Vibrio parahaemolyticus 2011VPH2	pVPH2	KP791968.1	156159	156815	+	VPA06_0196	qnrVC6_1	1-657/657	0/0	100	100	KC202804
Vibrio parahaemolyticus 20160303005-1	pVPSD2016-2	CP034301.1	131785	132176	+	VPA33_0142	bleO_1	8-399/399	0/0	98,25	100	AF051917
Vibrio parahaemolyticus 20160303005-1	pVPSD2016-2	CP034301.1	134862	135722	+	VPA33_0146	blaTEM-1B_1	1-861/861	0/0	100	100	AY458016
Vibrio parahaemolyticus 20160303005-1	pVPSD2016-2	CP034301.1	140857	142074	+	VPA33_0150	tet(E)_3	1-1218/1218	0/0	100	99,26	CP000645
Vibrio parahaemolyticus 20160303005-1	pVPSD2016-2	CP034301.1	145126	145992	-	VPA33_0154	sul1_5	1-867/867	0/0	100	99,89	EU780013
Vibrio parahaemolyticus 20160303005-1	pVPSD2016-2	CP034301.1	146423	147268	-	VPA33_0156	aadA16_1	1-846/846	0/0	100	99,64	EU675686
Vibrio parahaemolyticus 20160303005-1	pVPSD2016-2	CP034301.1	147449	147922	-	VPA33_0157	dfrA27_1	1-474/474	0/0	100	100	FJ459817
Vibrio parahaemolyticus 20160303005-1	pVPSD2016-2	CP034301.1	148055	148597	-	VPA33_0158	ARR-3_4	1-543/543	0/0	100	99,45	FM207631
Vibrio parahaemolyticus 20160303005-1	pVPSD2016-2	CP034301.1	153208	154413	+	VPA33_0162	tet(B)_1	1-1206/1206	0/0	100	99,83	AP000342
Vibrio parahaemolyticus NJIFDCVp7	pVP7-1	CP150866.1	144985	145376	-	VPA38_0150	bleO_1	8-399/399	0/0	98,25	100	AF051917

Strain	Plasmid	Plasmid ID	Start	End	Strand	Locus tag	Gene	Coverage	Gaps	%Coverage	%Identity	Accession
Vibrio parahaemolyticus NJIFDCVp7	pVP7-1	CP150866.1	147204	148064	+	VPA38_0153	blaTEM-1B_1	1-861/861	0/0	100	100	AY458016
Vibrio parahaemolyticus NJIFDCVp7	pVP7-1	CP150866.1	166060	167277	+	VPA38_0167	tet(E)_3	1-1218/1218	0/0	100	99,26	CP000645
Vibrio parahaemolyticus NJIFDCVp7	pVP7-1	CP150866.1	170329	171195	-	VPA38_0171	sul1_5	1-867/867	0/0	100	99,89	EU780013
Vibrio parahaemolyticus NJIFDCVp7	pVP7-1	CP150866.1	171459	172258	+	VPA38_0172	blaTEM-1B_1	62-861/861	0/0	92,92	100	AY458016
Vibrio parahaemolyticus NJIFDCVp7	pVP7-1	CP150866.1	177393	178610	+	VPA38_0176	tet(E)_3	1-1218/1218	0/0	100	99,26	CP000645
Vibrio parahaemolyticus NJIFDCVp7	pVP7-1	CP150866.1	181662	182528	-	VPA38_0180	sul1_5	1-867/867	0/0	100	99,89	EU780013
Vibrio parahaemolyticus NJIFDCVp7	pVP7-1	CP150866.1	182959	183804	-	VPA38_0182	aadA16_1	1-846/846	0/0	100	99,64	EU675686
Vibrio parahaemolyticus NJIFDCVp7	pVP7-1	CP150866.1	183985	184458	-	VPA38_0183	dfrA27_1	1-474/474	0/0	100	100	FJ459817
Vibrio parahaemolyticus NJIFDCVp7	pVP7-1	CP150866.1	184591	185133	-	VPA38_0184	ARR-3_4	1-543/543	0/0	100	99,45	FM207631
Vibrio parahaemolyticus NJIFDCVp7	pVP7-1	CP150866.1	189744	190949	+	VPA38_0188	tet(B)_1	1-1206/1206	0/0	100	99,83	AP000342
Vibrio parahaemolyticus V36	pVPH1	KP688397.1	123045	123587	+	VPA12_0147	ARR-3_4	1-543/543	0/0	100	99,45	FM207631
Vibrio parahaemolyticus V36	pVPH1	KP688397.1	123720	124193	+	VPA12_0148	dfrA27_1	1-474/474	0/0	100	100	FJ459817
Vibrio parahaemolyticus V36	pVPH1	KP688397.1	124686	125552	+	VPA12_0150	sul1_5	1-867/867	0/0	100	99,89	EU780013
Vibrio parahaemolyticus V36	pVPH1	KP688397.1	127838	128764	+	VPA12_0152	blaPER-1_1	1-927/927	0/0	100	100	GU944725
Vibrio parahaemolyticus V36	pVPH1	KP688397.1	133683	134549	+	VPA12_0159	sul1_5	1-867/867	0/0	100	99,89	EU780013
Vibrio parahaemolyticus V36	pVPH1	KP688397.1	141171	142092	-	VPA12_0166	mph(A)_2	1-921/921	1/1	100	99,67	U36578
Vibrio parahaemolyticus Vb0677	pVb677-tet	OQ622008.1	22946	23602	+	VPA13_0025	qnrVC6_1	1-657/657	0/0	100	100	KC202804
Vibrio parahaemolyticus Vb0677	pVb677-tet	OQ622008.1	24228	24701	+	VPA13_0026	dfrA6_2	1-474/474	0/0	100	99,37	FJ905898
Vibrio parahaemolyticus Vb0677	pVb677-tet	OQ622008.1	121796	122611	+	VPA13_0147	sul2_2	1-816/816	0/0	100	100	AY034138
Vibrio parahaemolyticus Vb0677	pVb677-tet	OQ622008.1	122899	123770	+	VPA13_0148	mef(C)_1	1-872/1224	0/0	71,24	100	AB571865
Vibrio parahaemolyticus Vb0677	pVb677-tet	OQ622008.1	126931	128850	-	VPA13_0153	tet(M)_8	1-1920/1920	0/0	100	99,74	X04388
Vibrio parahaemolyticus Vb0677	pVb677-tet	OQ622008.1	131299	132504	-	VPA13_0159	tet(B)_1	1-1206/1206	0/0	100	99,92	AP000342
Vibrio parahaemolyticus vp-201806	pA-vp-201806	CP150859.1	14511	15167	-	VPA36_0016	qnrS5_1	1-657/657	0/0	100	95,13	HQ631377
Vibrio parahaemolyticus vp-201806	pA-vp-201806	CP150859.1	106998	107813	+	VPA36_0126	sul2_18	1-816/816	0/0	100	100	AJ830714
Vibrio_parahaemolyticus_Vp2015094	Vp2015094_p1	CP080480.1	22703	23359	+	VPA22_0023	qnrVC6_1	1-657/657	0/0	100	100	KC202804
Vibrio parahaemolyticus Vp2015094	Vp2015094_p1	CP080480.1	23985	24458	+	VPA22_0024	dfrA6_2	1-474/474	0/0	100	99,37	FJ905898
Vibrio parahaemolyticus Vp2015094	Vp2015094_p1	CP080480.1	110077	111290	+	VPA22_0127	floR_2	1-1214/1215	0/0	99,92	98,19	AF118107
Vibrio parahaemolyticus Vp2015094	Vp2015094_p1	CP080480.1	112247	113083	-	VPA22_0130	aph(6)-Id_1	1-837/837	0/0	100	100	M28829
Vibrio parahaemolyticus Vp2015094	Vp2015094_p1	CP080480.1	113083	113885	-	VPA22_0131	aph(3")-Ib_2	2-804/804	0/0	99,88	100	AF024602
Vibrio parahaemolyticus Vp2015094	Vp2015094_p1	CP080480.1	113947	114762	-	VPA22_0132	sul2_15	1-816/816	0/0	100	100	FJ968160
Vibrio parahaemolyticus Vp2015094	Vp2015094_p1	CP080480.1	143161	145080	-	VPA22_0165	tet(M)_8	1-1920/1920	0/0	100	99,74	X04388
Vibrio parahaemolyticus Vp2015094	Vp2015094_p1	CP080480.1	147735	148940	-	VPA22_0171	tet(B)_1	1-1206/1206	0/0	100	99,92	AP000342
Vibrio sp. 04Ya090	pAQU2	AB856327.1	111479	113398	-	VSP28_0137	tet(M)_8	1-1920/1920	0/0	100	99,74	X04388
Vibrio sp. 04Ya090	pAQU2	AB856327.1	116053	117258	-	VSP28_0144	tet(B)_1	1-1206/1206	0/0	100	99,83	AP000342
Vibrio cyclitrophicus IHPE 6718	pAQU-MAN_2	ERX14309695	109335	109976	-	VSP25_0128	catA2_1	1-642/642	0/0	100	89,56	X53796
Vibrio cyclitrophicus IHPE 6718	pAQU-MAN_2	ERX14309695	117605	118246	+	VSP25_0142	catA2_1	1-642/642	0/0	100	89,56	X53796
Vibrio cyclitrophicus IHPE 6718	pAQU-MAN_2	ERX14309695	127405	128610	+	VSP25_0151	tet(B)_1	1-1206/1206	0/0	100	99,83	AP000342

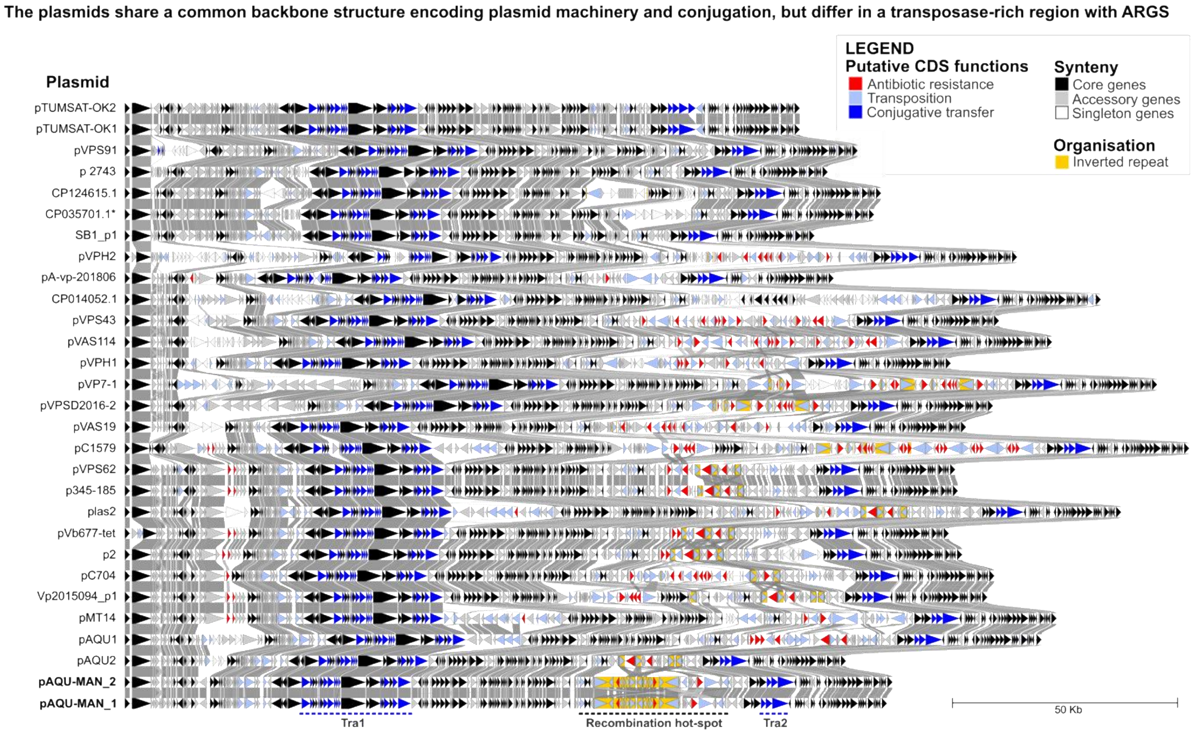

Figure S4: Synteny plots displaying genome structure of closely related plasmids, highlighting their similarities and differences. The clustering of protein families, considering 30% sequence identity and 50% coverage, resulted in 107 core genes (black), 314 accessory genes (grey), and 278 singleton genes (white). Genes are color-coded according to predicted function: red, antibiotic resistance; light blue, transposition; and cyan blue, conjugative transfer. The inverted repeat regions are shown in orange, with a minimum size of 100 bp and 75% identity between repeats.

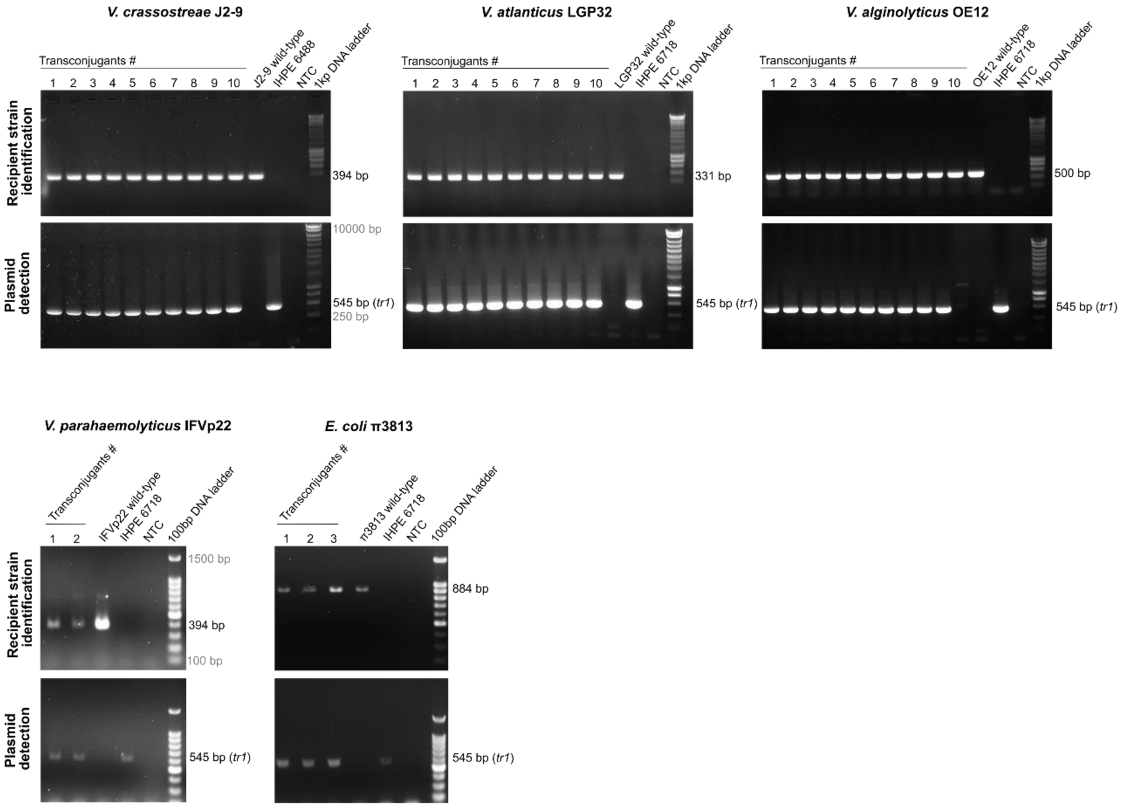

Figure S5: Recipient cells from the conjugation assay were confirmed to carry the R-plasmid. Agarose gel electrophoresis images of colony-PCR products are represented. Both strain identification (upper gel) and plasmid verification (lower gel) were performed by colony-PCR to confirm that the transconjugants were true recipient derivatives and not spontaneous chloramphenicol-resistant mutants. Between 3 and 10 transconjugants (when possible) were collected and analysed. Wild-type strains served as a positive control for recipient strain identification, while the donor strains (IHPE 6488 or IHPE 6718) were used as positive controls for plasmid detection. A No Template Control (NTC), free of DNA, was used as negative control. A 1kb or 100bp DNA ladder was used to confirm the size of the PCR products, visible on the right side of each gel.

Table S12: Whole-genome sequencing results

											qPCR	PCR		WG sequencing						
**Isolate Name**	**IHPE Name**	**Plasmid name**	**Pool**	**Date of collection**	**Hatchery time**	**Sample**	**Antibiotic treatment history**	**Tank name**	**Sampling number**	**Culture condition**	***tr2* gene**	***tr1* gene**	***catA2***	**Species identification**	**Accession**	**Sequencing technology**	**Total assembly lenghth**	**Number of contigs**	**Largest contig**	**GC %**
J20_EDM_11	IHPE 6229	/	-	08/02/2021	D-27	Seawater	A-	B3	91	TCBS 20°C	+	+	+	*V. crassostreae*	ERX14309691	Illumina	5282364	45	631352	44.29
J20_EDM_12	IHPE 6230	/	-	08/02/2021	D-27	Seawater	A-	B3	91	TCBS 20°C	+	+	+	*V. splendidus*	ERX14309670	Illumina	6235156	70	295828	43.58
J20_EDM_Cm_12	IHPE 6254	/	-	08/02/2021	D-27	Seawater	CHL	B1	89	TCBS 20°C	+	+	+	*V. crassostreae*	ERX14309678	Illumina	5319290	49	572891	44.3
J20_EDM_Cm_14	IHPE 6256	/	-	08/02/2021	D-27	Seawater	CHL	B7	93	TCBS 20°C	+	+	+	*V. splendidus*	ERX14309680	Illumina	5526503	70	507589	44.16
J20_EDM_Cm_15	IHPE 6257	/	-	08/02/2021	D-27	Seawater	CHL	B7	93	TCBS 20°C	+	+	+	*V. splendidus*	ERX14309690	Illumina	5435955	29	867772	44.26
J20_EDM_Cm_19	IHPE 6261	/	-	08/02/2021	D-27	Seawater	CHL	B7	93	TCBS 20°C	+	+	+	*V. splendidus*	ERX14309674	Illumina	6228072	96	339962	43.58
J20_EDM_Cm_20	IHPE 6262	/	-	08/02/2021	D-27	Seawater	CHL	B7	93	TCBS 20°C	+	+	+	*V. splendidus*	ERX14309677	Illumina	5358015	60	325280	44.17
J20_EDM_Cm_21	IHPE 6263	/	-	08/02/2021	D-27	Seawater	CHL	B7	93	TCBS 20°C	+	+	+	*V. splendidus*	ERX14309692	Illumina	5348460	84	378862	44.18
J20_EDM_Cm_22	IHPE 6264	/	-	08/02/2021	D-27	Seawater	CHL	B7	93	TCBS 20°C	+	+	+	*V. splendidus*	ERX14309672	Illumina	5404911	79	416250	44.17
J20_EDM_Cm_23	IHPE 6265	/	-	08/02/2021	D-27	Seawater	CHL	B7	93	TCBS 20°C	+	+	+	*V. splendidus*	ERX14309669	Illumina	5570079	354	378862	44.15
J34_EDM_Cm_03	IHPE 6323	/	-	22/02/2021	D-13	Seawater	CHL	B1	113	TCBS 20°C	+	+	+	*V. splendidus*	ERX14309687	Illumina	5580580	287	807025	44.23
J34_EDM_Cm_09	IHPE 6329	/	-	22/02/2021	D-13	Seawater	CHL	B7	117	TCBS 20°C	+	+	+	*V. splendidus*	ERX14309686	Illumina	5982695	455	421118	44.11
J34_EDM_Cm_10	IHPE 6330	/	-	22/02/2021	D-13	Seawater	CHL	B7	117	TCBS 20°C	+	+	+	*V. splendidus*	ERX14309682	Illumina	5428929	317	851770	44.12
J2R_LD_01	IHPE 6494	/	-	10/03/2021	D+2	Larvae	CHL	B1	152	TCBS 20°C	+	+	+	*V. cyclitrophicus*	ERX14309689	Illumina	5178504	327	758834	43.58
J2R_LD_04	IHPE 6497	/	-	10/03/2021	D+2	Larvae	CHL	B1	152	TCBS 20°C	+	+	+	*V. cyclitrophicus*	ERX14309684	Illumina	5170383	330	760324	43.59
J2R_LD_48	IHPE 6541	/	-	10/03/2021	D+2	Larvae	CHL	B7	160	TCBS 20°C	+	+	+	*V. splendidus*	ERX14309688	Illumina	5611774	343	698436	44.24
J2R_LD_53	IHPE 6546	/	-	10/03/2021	D+2	Larvae	CHL	B7	160	TCBS 20°C	+	+	+	*V. splendidus*	ERX14309679	Illumina	5503034	378	726920	44.18
J2R_LD_63	IHPE 6556	/	-	10/03/2021	D+2	Larvae	A-	B8	162	TCBS 20°C	+	+	+	*V. cyclitrophicus*	ERX14309673	Illumina	5172034	280	758834	43.59

											qPCR	PCR		WG sequencing						
**Isolate Name**	**IHPE Name**	**Plasmid name**	**Pool**	**Date of collection**	**Hatchery time**	**Sample**	**Antibiotic treatment history**	**Tank name**	**Sampling number**	**Culture condition**	***tr2* gene**	***tr1* gene**	***catA2***	**Species identification**	**Accession**	**Sequencing technology**	**Total assembly lenghth**	**Number of contigs**	**Largest contig**	**GC %**
J2R_LD_65	IHPE 6558	/	-	10/03/2021	D+2	Larvae	A-	B8	162	TCBS 20°C	+	+	+	*V. splendidus*	ERX14 309681	Illumina	5503650	407	727045	44.18
J7_L_21	IHPE 6690	/	-	15/03/2021	D+7	Larvae	A- /FLU	L2 (from B8)	169a	TCBS 20°C	+	+	+	*V. splendidus*	ERX14 309685	Illumina	5612862	355	665397	44.17
J7_L_28	IHPE 6697	/	-	15/03/2021	D+7	Larvae	CHL/ FLU	L5 (from B7)	172a	TCBS 20°C	+	+	+	*V. splendidus*	ERX14 309683	Illumina	5814459	341	479172	44.12
J7_L_32	IHPE 6701	/	-	15/03/2021	D+7	Larvae	CHL/ FLU	L5 (from B7)	172a	TCBS 20°C	+	+	+	*V. splendidus*	ERX14 309676	Illumina	5816177	353	755262	44.12
J7_L_46	IHPE 6715	/	-	15/03/2021	D+7	Larvae	CHL/ A-	L7 (from B1)	174a	TCBS 20°C	+	+	+	*V. cyclitrophicus*	ERX14 309693	Illumina	5171188	306	758834	43.59
J15R_L_24	IHPE 6798	/	-	23/03/2021	D+15	Larvae	CHL/ FLU	L5 (from B7)	229a	TCBS 20°C	+	+	+	*V. lentus*	ERX14 309671	Illumina	5781370	264	931817	44.07
J15R_L_35	IHPE 6809	/	-	23/03/2021	D+15	Larvae	A- /FLO	L3 (from B8)	221 b	TCBS 20°C	+	+	+	*V. splendidus*	ERX14 309675	Illumina	5621272	376	686037	44.22
J47_SPOVO_CmR_ 01	IHPE 6488	pAQU-MAN_1	1	08/03/2021	D0	Sperm	CHL	B1	129a	TCBS + CHL 10µg/ml 20°C	+	+	+	*V. corallirubri*	ERX14 309694	MinION	5300333	4	3405275	43,7
J7R_L_CmR_01	IHPE 6718	pAQU-MAN_2	4	15/03/2021	D+7	Larvae	A- /FLU	L2 (from B8)	169a	TCBS + CHL 10µg/ml 20°C	+	+	+	*V. cyclitrophicus*	ERX14 309695	MinION	5989745	5	3618762	44,44

3. Supplemental References

1. Petton B, Pernet F, Robert R, et al. Temperature influence on pathogen transmission and subsequent mortalities in juvenile Pacific oysters *Crassostrea gigas*. *Aquac Environ Interact*. 2013;3(3):257-273. doi:10.3354/aei00070

2. Petton B, Bruto M, James A, et al. *Crassostrea gigas* mortality in France: the usual suspect, a herpes virus, may not be the killer in this polymicrobial opportunistic disease. *Front Microbiol*. 2015;6:149878. doi:10.3389/fmicb.2015.00686

3. De Gregoris TB, Aldred N, Clare AS, et al. Improvement of phylum-and class-specific primers for real-time PCR quantification of bacterial taxa. *J Microbiol Methods*. 2011;86(3):351-356. doi:10.1016/j.mimet.2011.06.010

4. Bekal S, Brousseau R, Masson L, et al. Rapid identification of *Escherichia coli* pathotypes by virulence gene detection with DNA microarrays. *J Clin Microbiol*. 2003;41(5):2113-2125. doi:10.1128/jcm.41.5.2113-2125.2003

5. Lemire A, Goudenège D, Versigny T, et al. Populations, not clones, are the unit of vibrio pathogenesis in naturally infected oysters. *ISME J*. 2015;9(7):1523-1531. doi:10.1038/ismej.2014.233

6. Le Roux F, Zouine M, Chakroun N, et al. Genome sequence of *Vibrio splendidus*: an abundant planctonic marine species with a large genotypic diversity. *Environ Microbiol*. 2009;11(8):1959-1970. doi:10.1111/j.1462-2920.2009.01918.x

7. Gay M, Berthe FC, Le Roux F. Screening of *Vibrio* isolates to develop an experimental infection model in the Pacific oyster *Crassostrea gigas*. *Dis Aquat Org*. 2004;59(1):49-56. doi:10.3354/dao059049

8. Sorée M, Lozach S, Kéomurdjian N, et al. Virulence phenotypes differ between toxigenic *Vibrio parahaemolyticus* isolated from western coasts of Europe. *Microbiol Res*. 2024;285:127744. doi:10.1016/j.micres.2024.127744

9. Le Roux F, Binesse J, Saulnier D, et al. Construction of a *Vibrio splendidus* mutant lacking the metalloprotease gene vsm by use of a novel counterselectable suicide vector. *Appl Environ Microbiol*. 2007;73(3):777-784. doi:10.1128/AEM.02147-06
